# Furosemide stress test as a predictive marker of acute kidney injury progression or renal replacement therapy: a systemic review and meta-analysis

**DOI:** 10.1186/s13054-020-02912-8

**Published:** 2020-05-07

**Authors:** Jia-Jin Chen, Chih-Hsiang Chang, Yen-Ta Huang, George Kuo

**Affiliations:** 1 Department of Nephrology, Chang Gung Memorial Hospital, No 5 Fu-shin Street, Taoyuan City, 333 Taiwan; 2 Department of Nephrology, Kidney Research Center, Chang Gung Memorial Hospital, Taoyuan City, 333 Taiwan; 3 Division of Experimental Surgery, Department of Surgery, Buddhist Tzu Chi General Hospital, Hualien County, 970 Taiwan; 4 Department of Pharmacology, School of Medicine, Tzu Chi University, Hualien County, 970 Taiwan

**Keywords:** Furosemide stress test, Acute kidney injury, Severity prediction

## Abstract

**Background:**

The use of the furosemide stress test (FST) as an acute kidney injury (AKI) severity marker has been described in several trials. However, the diagnostic performance of the FST in predicting AKI progression has not yet been fully discussed.

**Methods:**

In accordance with the Preferred Reporting Items for Systematic Reviews and Meta-Analyses (PRISMA) guidelines, we searched the PubMed, Embase, and Cochrane databases up to March 2020. The diagnostic performance of the FST (in terms of sensitivity, specificity, number of events, true positive, false positive) was extracted and evaluated.

**Results:**

We identified eleven trials that enrolled a total of 1366 patients, including 517 patients and 1017 patients for whom the outcomes in terms of AKI stage progression and renal replacement therapy (RRT), respectively, were reported. The pooled sensitivity and specificity results of the FST for AKI progression prediction were 0.81 (95% CI 0.74–0.87) and 0.88 (95% CI 0.82–0.92), respectively. The pooled positive likelihood ratio (LR) was 5.45 (95% CI 3.96–7.50), the pooled negative LR was 0.26 (95% CI 0.19–0.36), and the pooled diagnostic odds ratio (DOR) was 29.69 (95% CI 17.00–51.85). The summary receiver operating characteristics (SROC) with pooled diagnostic accuracy was 0.88. The diagnostic performance of the FST in predicting AKI progression was not affected by different AKI criteria or underlying chronic kidney disease. The pooled sensitivity and specificity results of the FST for RRT prediction were 0.84 (95% CI 0.72–0.91) and 0.77 (95% CI 0.64–0.87), respectively. The pooled positive LR and pooled negative LR were 3.16 (95% CI 2.06–4.86) and 0.25 (95% CI 0.14–0.44), respectively. The pooled diagnostic odds ratio (DOR) was 13.59 (95% CI 5.74–32.17), and SROC with pooled diagnostic accuracy was 0.86. The diagnostic performance of FST for RRT prediction is better in stage 1–2 AKI compared to stage 3 AKI (relative DOR 5.75, 95% CI 2.51–13.33).

**Conclusion:**

The FST is a simple tool for the identification of AKI populations at high risk of AKI progression and the need for RRT, and the diagnostic performance of FST in RRT prediction is better in early AKI population.

## Introduction

The incidence of in-hospital acute kidney injury (AKI), depending on the different AKI criteria used, ranges from 7.0–18.3% [1] among hospitalized patients in general and up to 20–50% in critically ill populations [2]. The progression of AKI with multiple organ failure can result in poor prognosis. Because of the high morbidities and mortalities associated with AKI, many investigators have focused on several novel biomarkers for earlier detection of AKI, discrimination of etiologies, and prediction of outcomes [3–7]. However, the availability of these novel biomarkers may be limited by its expense or reimbursement issues in different countries. In addition to the therapeutic role of furosemide on fluid balance, blood pressure control, and the management of hypercalcemia, Chawla et al. proposed furosemide stress test (FST) as a tool for predicting AKI progression [8]. Several following studies also utilized FST to predict AKI progression or RRT prediction, but with heterogeneity in AKI criteria, cutoff value of urine output, duration of monitor, or study designs. A few recent studies used FST to predict delayed graft function after kidney transplant [9, 10], and others focused on child populations [11, 12]. As such, in order to more effectively explore the diagnostic accuracy of the FST to predict AKI progression and renal replacement therapy (RRT) initiation, we conducted this meta-analysis according to the Preferred Reporting Items for Systematic Reviews and Meta-Analyses (PRISMA) diagnostic test accuracy guidelines [13].

## Methods

### Literature search

In accordance with the PRISMA guidelines, two investigators (JJ-C, G-K) systematically and independently conducted a review of the relevant published data. A computerized search of the Pubmed, Embase, and Cochrane electronic databases was performed using the keywords “furosemide,” “acute kidney injury,” “acute kidney failure,” and “renal insufficiency,” and medical subject heading (MeSH) terms “Furosemide” [Mesh], “renal insufficiency”[Mesh] AND “Acute Kidney Injury” [Mesh] in order to identify all the relevant studies up to March 2020. Review articles or meta-analyses were not included for analysis, but their citations and references were searched for additional relevant studies. The detail results of literature search were provided in Additional profile 1: Supplementary Table 1A and 1B. We also performed search of gray literature, and the detail is provided in Additional profile 2: Supplementary document.

### Study selection

After the initial screening, the two investigators Jia Jin Chen (JJ-C) and George Kuo (G-K) independently determined the eligibility of the identified studies based on evaluations of their titles, abstracts, and, subsequently, full texts. Any differences in opinion regarding eligibility were resolved by consensus through discussion with Chih-Hsiang Chang. The full text of any article that was deemed potentially relevant was retrieved online. A study was included if it met the criteria of adult humans as its population, and reported the protocol and cutoff point of the FST. We enrolled studies with primary or secondary outcomes reporting the diagnostic value of the FST for AKI progression, RRT, or mortality. Studies were excluded if they met one or more of the following criteria: (1) focused on a population with solid organ or hematopoietic stem cell transplantation, (2) used duplicate cohorts, (3) contained insufficient information for analysis, (4) included pediatric patients, or (5) did not report outcome of interest. Detailed results regarding excluded studies and the reasons for their exclusion are available in Additional profile 1: Supplementary Table 2. We have registered our work in PROSPERO. However, till we finished our work, the registration was still under assessed by the editorial team of PROSPERO; therefore, we provided our initial registered protocol as Additional profile 3.

### Data extraction

The two investigators independently extracted relevant information from each study. The extracted data elements included the first author, year of publication, study location, study design, diagnostic criteria of AKI, total sample size, protocol of the FST (that is, furosemide dose, duration of monitor, cutoff value of urine output), patients’ AKI stages, outcomes of interest, whether or not the enrolled population had high plasma neutrophil gelatinase-associated lipocalin (NGAL) levels, and whether patients with chronic kidney disease were excluded or not (Table 1). As for diagnostic test performance, the extracted data included the cutoff value of urine output based on the Youden index or pre-defined criteria, sensitivity, specificity, number of true positive, number of false positive, and the event number of AKI progression, RRT, or mortality (Table 1 and Table 2).

**Table 1:** The characteristics of the eleven included studies

First author/year	Location	Design	AKI criteria	Population	Sample size	Furosemide dose	Urine output cutoff point	Outcome of interest	Enrolled patients AKI stage	High plasma NGAL	Exclusion of chronic kidney disease
Chawla, 2013 [8]	USA	PC+RC	AKIN	Mixed	77	1 mg/kg (furosemide naïve) or 1.5 mg/kg (furosemide non-naive)	200 ml/2 h	AKIN stage 3	AKIN stage 1–2	Yes	Yes (eGFR < 30)
Elsaegh, 2018 [14]	Egypt	PC	KDIGO	Sepsis	60			KDIGO stage progression (included RRT)	Normal renal function and any stage of AKI	No	Yes
Lumlertgul, 2018 [15]	Thailand	PC	KDIGO	Mixed	162			RRT	Any stage of AKI	Yes	Yes (baseline Cr > 2)
Martínez, 2016 [16]	Mexico	PC	KDGO	NA	20			KDIGO stage-3 and RRT	KDIGO stage 1–2	No	Yes (eGFR < 30)
Matsuura, 2018 [17]	Japan	RC	KDIGO	Mixed	51	NA	3.9 ml/2 h for per milligram furosemide	KDIGO stage 3 and RRT	KDIGO stage 1–2 or high NGAL with normal renal function	Yes	No
Pérez-Cruz, 2017 [18]	Mexico	PC	AKIN	Medical	35	1 mg/kg (furosemide naive) or 1.5 mg/kg (furosemide non-naive)	200 ml/2 h	AKIN-3 and RRT	AKIN stage 1–2	No	No
Rewa, 2019 [19]	USA and Canada	PC	AKIN	Mixed	92			AKIN stage 3	AKIN stage 1–2	No	Yes (eGFR < 30)
Saber, 2018 [20]	Egypt	PC	AKIN	NA	40		325 ml/6 h	AKIN stage 3 (included RRT)	AKIN stage 1–2	No	Yes (eGFR < 30)
Sakhuja, 2019 [21]	USA	RC	AKIN	NA	687	≥ 1 mg/kg	600 ml/6 h	RRT	AKIN stage 3	No	No
Vairakkani, 2019 [22]	India	NA	KDIGO	NA	80	1 mg/kg (furosemide naive) or 1.5 mg/kg (furosemide non-naive)	325 ml/2 h	KDIGO stage 3	KDIGO stage 1–2	No	Yes (eGFR < 30)
Venugopal, 2019 [23]	India	PC	AKIN	NA	62		200 ml/2 h	AKIN-3 and RRT	AKIN stage 1–2	No	No

*Abbreviation*: *AKI* acute kidney injury, *AKIN* Acute Kidney Injury Network, *Cr* creatinine, *eGFR* estimated glomerular filtration rate, *KDIGO* Kidney Disease Global outcomes, *NA* not applicable, *PC* prospective cohort, *RC* retrospective cohort, *RRT* renal replacement therapy

**Table 2:** Diagnostic test performance of furosemide stress test for AKI progression, renal replacement therapy, and mortality

Study	Sensitivity	Specificity	Sample size	Event (AKI progression)	TP	FP	FN	TN	Follow-up period
Chawla, 2013 [8]	87.1	84.1	77	25	22	8	3	44	14 days
Elsaegh, 2018 [14]	89.3	93.4	60	28	25	2	3	30	NA
Martínez, 2016 [16]	66.7	100	20	6	4	0	2	14	30 days
Matsuura, 2018 [17]	76.5	94.1	51	17	13	2	4	32	7 days
Pérez-Cruz, 2017 [18]	57.1	95.2	35	14	8	1	6	20	NA
Saber, 2018 [20]	86.7	68	40	15	13	8	2	17	NA
Rewa, 2019 [19]	73.9	90	92	23	17	7	6	62	30 days
Vairakkani, 2019 [22]	82	80.8	80	28	23	10	5	42	14 days
Venugopal, 2019 [24]	85.7	87.5	62	14	12	6	2	42	NA
Study	Sensitivity	Specificity	Sample size	Event (RRT)	TP	FP	FN	TN	Follow-up period
Lumlertgul, 2018 [15]	94.4	70.4	162	108	102	16	6	38	28 days
Martínez, 2016 [16]	75	93.7	20	4	3	1	1	15	30 days
Matsuura, 2018 [17]	75	79	51	8	6	9	2	34	7 days
Pérez-Cruz, 2017 [18]	62.5	85.2	35	8	5	4	3	23	NA
Sakhuja, 2019 [21]	80.9	50.5	687	162	131	260	31	265	1 day
Venugopal, 2019 [23]	83.3	84	62	12	10	8	2	42	NA
Study	Sensitivity	Specificity	Sample size	Event (mortality)	TP	FP	FN	TN	Follow-up period
Martínez, 2016 [16]	20	80	20	5	1	3	4	12	30 days
Venugopal, 2019 [23]	66.7	77.3	62	9	6	12	3	41	NA

*Abbreviation:* AUROC area under the receiver operating characteristics, *AKI* acute kidney injury, *FN* false negative, *FP* false positive, *NA* not applicable, *RRT* renal replacement therapy, *TN* true negative, *TP* true positive

### Outcome measures

The diagnostic criteria for AKI were different in the eleven enrolled studies. Five of the studies (Elsaegh, Lumlertgul, Martínez, Matsuura, Vairakkani) [14–17, 22] used the Kidney Disease: Improving Global Outcomes (KDIGO) criteria [25]. Other studies used the Acute Kidney Injury Network (AKIN) criteria [26]. The reference test used in each study was based on the different AKI criteria in each trial or on whether the patients received RRT or mortality during the follow-up period. Four studies (Chawla, Pérez-Cruz, Rewa, Venugopal) [8, 18, 19, 23] used the AKIN stage 3 AKI as primary outcome. Three studies (Martínez, Matsuura, Vairakkani) [16, 17, 22] used the KDIGO stage 3 AKI as primary outcome. Two studies (Elsaegh, Saber) reported primary composite outcome consist of AKI progression and RRT [14, 20]. Six studies (Martínez, Lumlertgul, Matsuura, Pérez-Cruz, Sakhuja, Venugopal) reported outcome of RRT, and two studies (Martínez, Venugopal) reported outcome of mortality [15–18, 21, 23] (Table 2). Most studies reporting outcome of RRT did not mention the indications of renal replacement therapy except one (Lumlertgul) [15]. In this study, the patient received RRT within 6 h after randomization in early group or received RRT based on conventional indications in standard group.

### Risk of bias assessment

The risk of bias for each of the included studies was assessed using the Quality Assessment of Diagnostic Accuracy Studies 2 (QUADAS-2) tool and Review Manager version 5.3 to identify the quality of the included studies [27]. The QUADAS-2 tool is based on four domains (patient selection, index test, reference standard, and flow and timing), which are used to judge the risk of bias. Each study was reviewed independently by JJ-C and G-K, with each investigator assigning a rating of high, low, or unclear risk for all four domains. The judgment principle of “applicability” was the same as the bias section, but there were no signaling questions. Disagreements between the reviewers were resolved by discussion with another author, Chih-Hsiang Chang. If the answer to all the signaling questions for a given domain was “yes,” then the domain was considered to entail a low risk of bias. If the answer to any of the signaling questions for a domain was “no,” then the domain was considered to entail a high risk of bias. The quality of evidence for the diagnostic performance of the FST in this meta-analysis was assessed based on the guidelines of the GRADE Working Group methodology [28]. We summarized the results in a table, which was constructed using the online GRADE Profiler (see Additional profile 4).

### Statistical analysis

We extracted the event number, total sample size, and true positive (TP), true negative (TN), false positive (FP), and false negative (FN) rates for each study or calculated these values according to the reported sensitivity and specificity. Based on these data, the positive likelihood ratio (+LR), negative likelihood ratio (−LR), and diagnostic odds ratio (DOR) could be obtained for each study. The summary measures were calculated using a bivariate model for the pooled sensitivity and specificity. We used a random-effects model with maximum likelihood estimation to calculate the pooled DOR and LR. The above two tests were conducted by the “metabin” function in the “meta” package [29]. To assess the diagnostic performance of the FST regarding AKI progression for FST non-responders, a summary receiver operating characteristics (SROC) curve was constructed by the “restima” function with restricted maximum likelihood estimation in the “mada” package [24]. The threshold effect was examined by using the Spearman correlation coefficient between the logit of sensitivity and logit of “1 – specificity,” and *P* < 0.05 indicated the existence of a threshold effect. If there is no significant threshold effect, subgroup analysis or meta-regression analysis is warranted to clarify the sources of heterogeneity [30]. Heterogeneity from covariates other than the threshold effect among studies was evaluated using the *I*^2^ index, with *I*^2^ < 25%, 25–50%, and > 50% indicating mild, moderate, and high heterogeneity, respectively. The LRs indicate whether the accuracy of a particular test would be more accurate for patients with a disease than for subjects without the disease. Several relevant variables were identified, and these variables are summarized in Table 1, Table 2, and Additional profile 1: Supplementary Table 3 (with the specific variables including the diagnostic criteria of AKI, whether the enrolled patients had high plasma NGAL, whether or not the enrolled patients had a clinical diagnosis of AKI, the use of a pre-specified cutoff value of urine output, the used FST protocol, prospective or retrospective study design, and whether the patients with chronic kidney disease were excluded). To explore possible sources of heterogeneity, these variables were applied as moderators in meta-regression weighted by the inverse of the study variance. We performed the meta-regression by using “metareg” function in the “meta” package. A sensitivity analysis was performed after excluding studies using a composite outcome consist of AKI progression and RRT. All analyses were conducted using R version 3.6.2 (2019-12-12) [31]. A two-sided *P* value of < 0.05 was considered statistically significant.

## Results

### Literature search

**Fig. 1:**
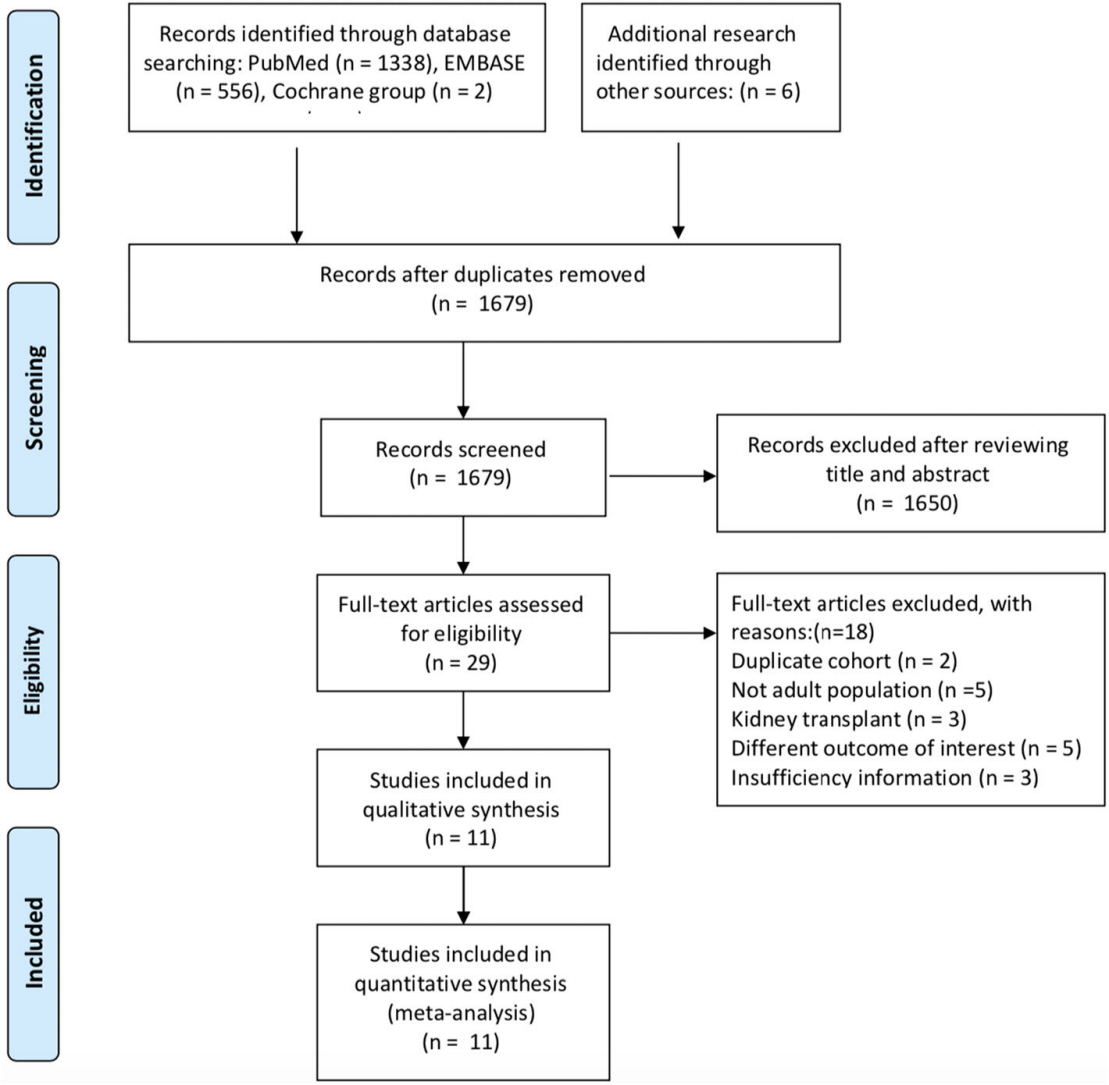
PRISMA flow chart of study inclusion

The initial search retrieved 1902 records. After excluding duplicate articles, the remaining 1679 articles were screened based on their titles and abstracts in order to identify the potentially relevant articles, the full texts of which then were downloaded and reviewed to further determine their eligibility for inclusion in the final analysis. Of the 29 articles, two [32, 33] were suspected of using a duplicate cohort from another study [8], five were focused on child populations [11, 12, 34–36], and three were based on kidney transplant outcomes [9, 10, 37]. Meanwhile, five studies reported different outcomes of interest and the remaining three did not report sufficient information for analysis [38–45] (Additional profile 1: Supplementary Table 2). As such, eleven studies were ultimately included in this meta-analytic study (Fig. 1).

### Study characteristics

The eleven included trials enrolled a total of 1366 patients with clinical AKI or a risk of AKI. Among those patients, 517 patients and 1017 patients, respectively, had reported outcomes of AKI progression (including the need for RRT) or RRT. Most of the enrolled studies used prospective cohorts, and the remaining four studies used non-prospective study designs or insufficient information about study designs (Chawla, Matsuura, Sakhuja, Vairakkani) [8, 17, 21, 22]. All of the studies, except the two by Matsuura et al. and Sakhuja et al., used a standard furosemide dose, which is 1 mg/kg for the furosemide naive patients and 1.5 mg/kg for those patients exposed to furosemide within 7 days prior to FST [17, 21]. Matsuura et al. used a complex cutoff value, which presented as urine volume divided by the administered furosemide dose (specifically, 3.9 ml of urine output 2 h after per milligram of furosemide administration) [17]. In the study by Sakhuja et al., the used dose of furosemide was at least 1 mg/kg [21]. Most of the studies used a 2-h time interval to determine the FST responsiveness; only one study (Saber) used a 6-h time interval [20]. Most studies used 200 ml urine output within 2 h after furosemide stress test as cutoff value except four studies (Matsuura; Saber; Sakhuja; Vairakkani) [17, 20–22] (Table 1). Three studies enrolled populations with high plasma NGAL levels (Chawla, Lumlertgul, Matsuura) [8, 15, 17]. Most studies did not report serum albumin level, which might be an important factor for diuresis response after furosemide administration. Only two studies reported serum albumin level (Matsuura average serum albumin 2.8 g/dl and Sakhuja average serum albumin level 2.9 g/dl) [17, 21]. Besides, the study by Lumlertgul et al. excluded patients with serum albumin level less than 2 g/dl [15] (Additional profile 1: Supplementary Table 3).

### Risk of bias

**Fig. 2:**
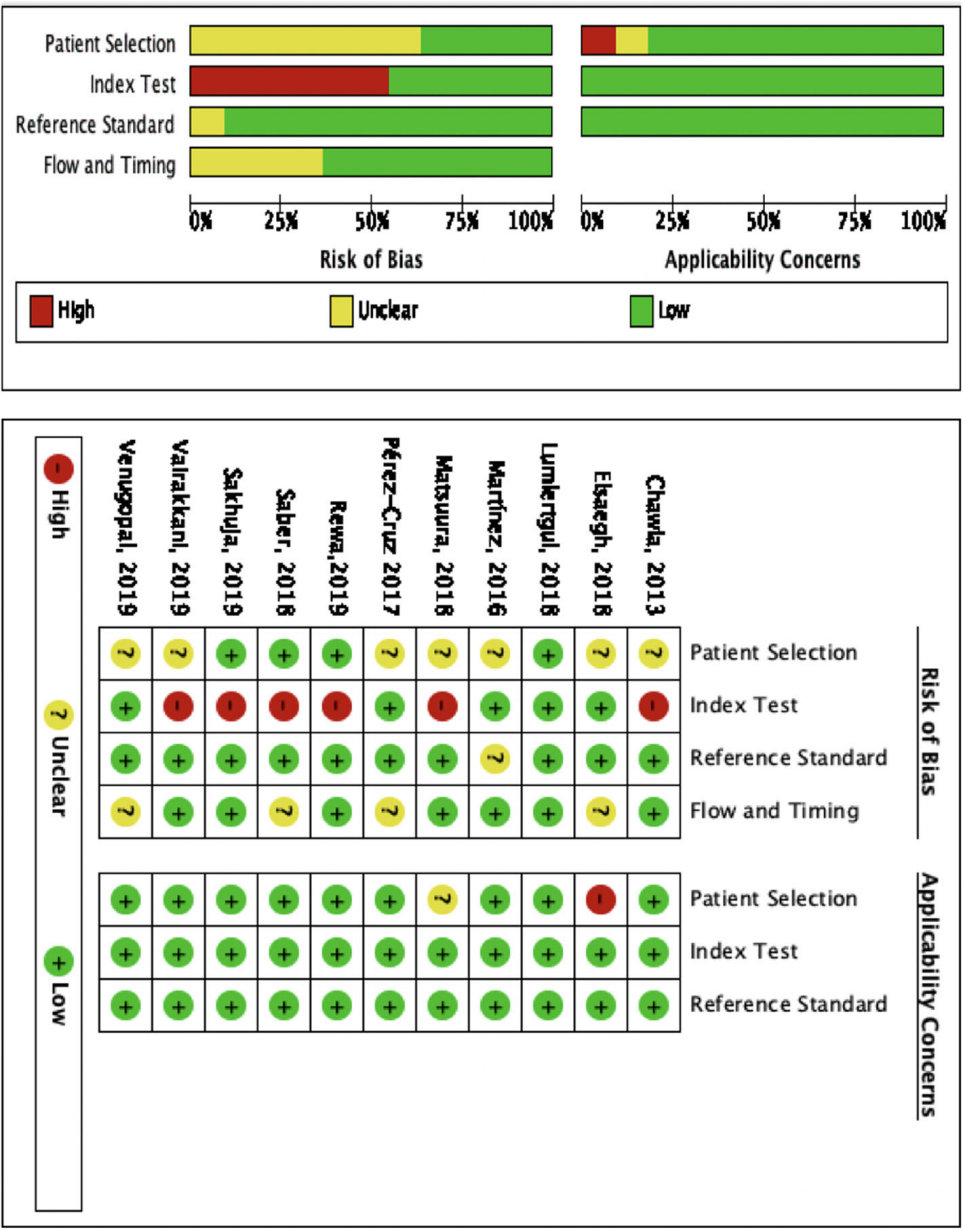
Summary of risk of bias and applicability concern

With the QUADAS-2 tool, study characteristics or designs that might increase the risk of bias were identified. Domain 1 of the QUADAS-2 tool focuses on patient selection. One study (Elsaegh) [14] enrolled septic ICU patients with normal renal function, and we considered this to entail a high risk of applicability concern. Another study (Matsuura) [17] enrolled patients with clinical AKI or subclinical AKI (that is, those with high biomarker levels that still did not meet the clinical AKI criteria). Two trials (Vairakkani, Venugopal) [22, 23] provided insufficient information about their study designs; therefore, the domain 1 aspects of the study populations for these two studies were considered to entail unclear risks. Domain 2 of the QUADAS-2 tool addresses the aspect of index tests. Six trials (Chawla; Matsuura; Rewa; Saber; Sakhuja; Vairakkani) [8, 17, 19–22] selected the urine output threshold to optimize sensitivity and/or specificity; therefore, these six studies were considered to have a high risk of bias regarding domain 2. All of the studies that used the AKIN or KDIGO AKI criteria or RRT as reference standard were considered to have low risk of bias. Four studies (Elsaegh; Pérez-Cruz; Saber; Venugopal) [14, 18, 20, 23] did not report a follow-up period for the primary or secondary outcomes. Therefore, these four studies were considered to have unclear risk of bias regarding domain 4. Because of the reasons mentioned above, we considered one study (Elsaegh) [14] to have high applicability concern regarding patient selection and another one (Matsuura) [17] to have unclear concern. The other two domains of applicability concern in the included studies were all rated as low risk. We conducted the risk of bias analysis for all the included studies using Review Manager (RevMan) version 5.3 [46], and the results are summarized in Fig. 2.

### Furosemide stress test for acute kidney injury stage progression prediction

**Fig. 3:**
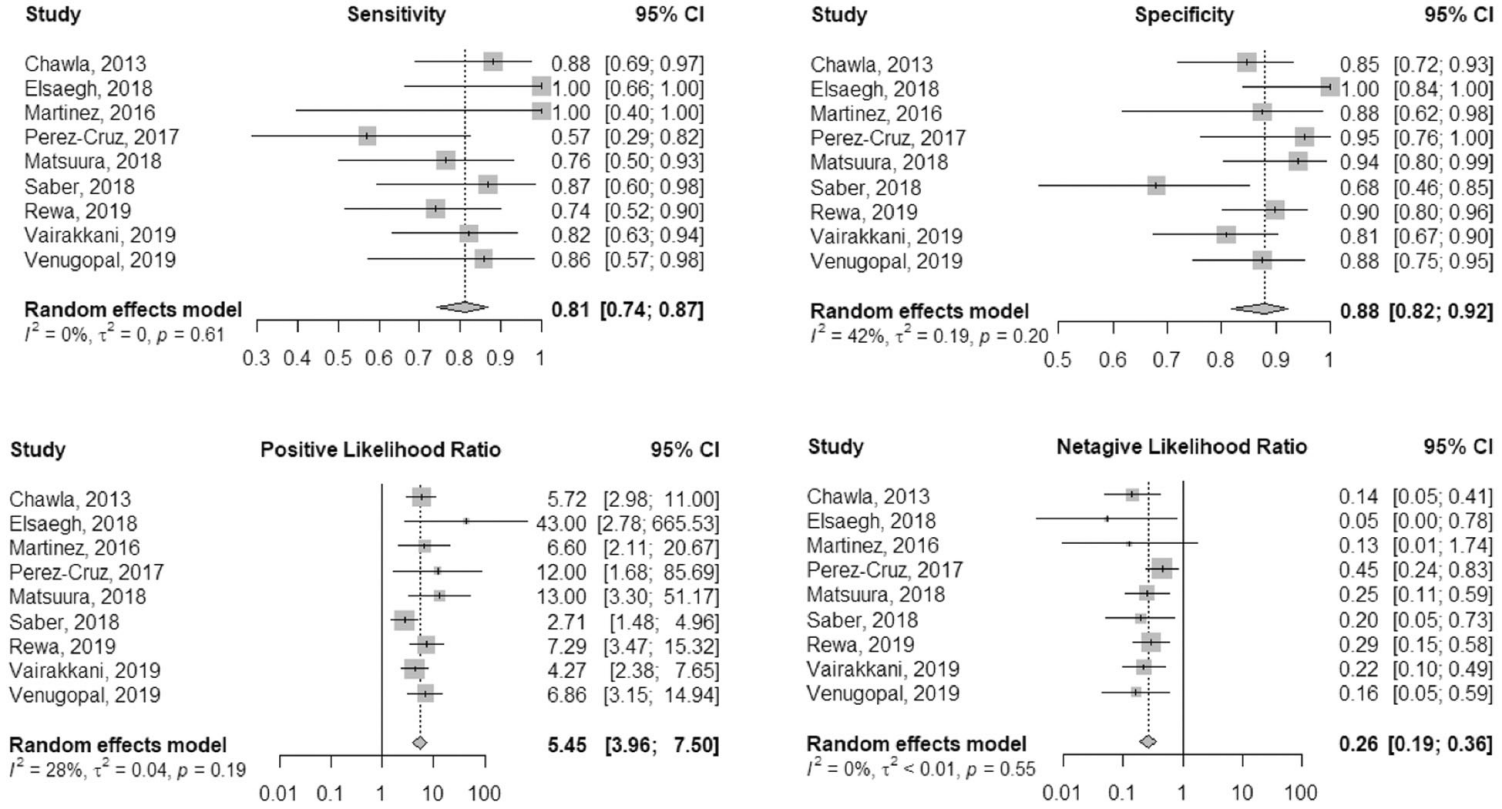
Forest plot of FST diagnostic accuracy for AKI progression prediction

The diagnostic values, cutoffs, and key results are summarized in Table 2. The pooled sensitivity and specificity values were 0.81 (95% CI 0.74–0.87) and 0.88 (95% CI 0.82–0.92), respectively. The pooled positive LR was 5.45 (95% CI 3.96–7.50), and the negative LR was 0.26 (95% CI 0.19–0.36) (Fig. 3). The heterogeneity of the aforementioned four pooled indices ranged from low to moderate (*I*^2^ ranged from 0.0 to 42%) (Fig. 3). The pooled DOR was 29.69 (95% CI 17.00–51.85), with low heterogeneity (*I*^2^ = 0) (Supplementary Fig. 1). The area under the curve (AUC) for SROC to summarize diagnostic accuracy was 0.88 (Supplementary Fig. 2).

### Furosemide stress test for renal replacement therapy prediction

**Fig. 4:**
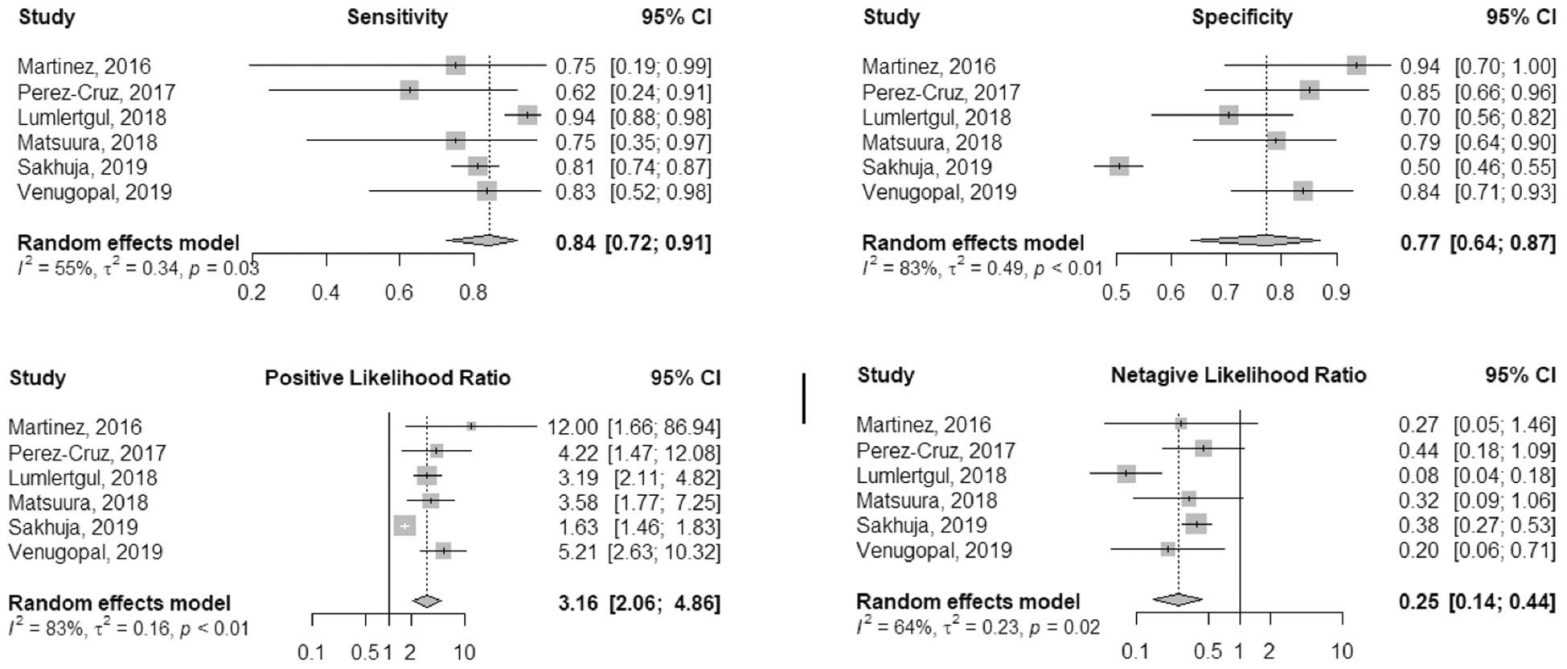
Forest plot of FST diagnostic accuracy for renal replacement therapy prediction

Six studies reported the diagnostic value of FST in predicting RRT in AKI populations. Four studies (Lumlertgul, Martínez, Pérez-Cruz, Venugopal) used FST protocol identical to that used by Chawla et al. (1 mg/kg for the furosemide-naive patients or 1.5 mg/kg for patients who have exposure to furosemide and 200 ml urine output after furosemide administration as cutoff value) [8, 15, 16, 18, 23]. One study (Matsuura) used complex cutoff value as abovementioned [17]. In one retrospective study (Sakhuja) [21], the patient received at least 1 mg/kg furosemide and the cutoff value of urine output was 600 ml at 6 h after FST (Table 1). The pooled sensitivity and specificity values were 0.84 (95% CI 0.72–0.91) and 0.77 (95% CI 0.64–0.87), respectively. The pooled positive LR was 3.16 (95% CI 2.06–4.86), and the negative LR was 0.25 (95% CI 0.14–0.44). The heterogeneity of the aforementioned four pooled indices was high (*I*^2^ ranged from 55 to 83%) (Fig. 4). The pooled DOR was 13.59 (95% CI 5.74–32.17), with high heterogeneity (*I*^2^ = 76%) (Supplementary Fig. 3). The area under the curve (AUC) for SROC to summarize diagnostic accuracy was 0.86 (Supplementary Fig. 4).

### Furosemide stress test for mortality prediction

Two studies (Martínez, Venugopal) reported the diagnostic value of FST for predicting mortality [16, 23]. Martínez et al. reported the prediction ability of FST for 30-day mortality. The follow-up period was unclear in the study by Venugopal et al. The pooled sensitivity and specificity values were 0.48 (95% CI 0.18–0.79) and 0.78 (95% CI 0.67–0.86), respectively. The pooled positive LR was 2.64 (95% CI 1.39–5.03), and the negative LR was 0.83 (95% CI 0.53–1.29) (Supplementary Figure 5). The heterogeneity of the aforementioned four pooled indices was low to high (*I*^2^ ranged from 0 to 58%). The pooled DOR was 4.09 (95% CI 1.11–15.12), with moderate heterogeneity (*I*^2^ = 38%) (Supplementary Figure 6). The area under the curve (AUC) for SROC to summarize diagnostic accuracy was 0.78 (Supplementary Figure 7).

### Subgroup analysis and sensitivity analysis

To explore the source of heterogeneity, we perform subgroup analysis in regard to the diagnostic criteria of AKI, prospective or non-prospective design, use of a pre-specified cutoff value of urine output, enrolled high NGAL population, different FST protocols, exclusion or inclusion of patients with baseline CKD, and whether the primary outcome was a pure outcome. The analysis of threshold effect was performed with Spearman rank correlations (*ρ* = 0.197; *P* = 0.62). The results implied that there was no significant threshold effect and subgroup analysis was required. The diagnostic performance of FST for AKI progression was not affected by different diagnostic criteria of AKI, exclusion or inclusion of CKD, different duration to monitor urine output, different FST protocol, or the purity of primary outcome. The results of the subgroup analysis and sensitivity analysis are summarized and presented in Table 3.

**Table 3:** Heterogeneity analysis of meta-analyses (outcome included sensitivity analysis and meta-regression) for furosemide stress test as an AKI progression prediction tool

Variables	Subgroups	Number of studies	Sensitivity (95% CI)	Specificity (95% CI)	Positive LR (95% CI)	Negative LR (95% CI)	Diagnostic odds ratio (95% CI)	Coeff.	SE	*P* value	RDOR (95% CI)
AKI criteria	AKIN	5	0.79 (0.68–0.87)	0.86 (0.79–0.91)	5.22 (3.45–7.89)	0.28 (0.19–0.42)	27.88 (13.85–56.12)	0.17	0.59	0.77	1.19 (0.37–3.78)
	KDIGO	4	0.84 (0.73–0.92)	0.91 (0.78–0.97)	5.66 (3.51–9.14)	0.21 (0.12–0.37)	33.11 (13.15–83.42)				
Cutoff point time	6 h	1						0.86	0.92	0.35	2.36 (0.38–14.30)
	2 h	8	0.81 (0.72–0.87)	0.89 (0.84–0.92)	6.23 (4.57–8.49)	0.27 (0.19–0.37)	32.52 (18.03–58.66)				
Study design	Non-prospective	3	0.83 (0.72–0.90)	0.86 (0.79–0.90)	5.32 (3.51–8.05)	0.21 (0.12–0.35)	30.03 (13.29–67.82)	− 0.02	0.57	0.97	0.98 (0.31–3.00)
	Prospective	6	0.82 (0.66–0.92)	0.89 (0.80–0.95)	5.70 (3.50–9.27)	0.30 (0.20–0.45)	29.39 (13.68–63.15)				
High NGAL	No	7	0.81 (0.70–0.89)	0.88 (0.80–0.94)	5.14 (3.54–7.46)	0.28 (0.20–0.41)	25.99 (13.66–49.44)	0.54	0.66	0.42	1.71 (0.46–6.23)
	Yes	2	0.83 (0.69 0.92)	0.88 (0.80–0.93)	6.66 (3.69–12.01)	0.20 (0.10–0.39)	44.42 (14.50–136.10)				
Exclusion of late CKD	No	3	0.73 (0.59–0.84)	0.91 (0.84–0.95)	8.37 (4.41–15.86)	0.33 (0.21–0.53)	40.84 (13.66–122.10)	− 0.43	0.65	0.51	0.65 (0.18–2.31)
	Yes	6	0.85 (0.76–0.90)	0.86 (0.78–0.91)	4.79 (3.42–6.71)	0.21 (0.14–0.33)	26.55 (13.89–50.75)				
FST protocol	Others	3	0.82 (0.70–0.90)	0.83 (0.68–0.92)	3.85 (2.57–5.74)	0.23 (0.13–0.39)	22.16 (9.38–52.32)	0.51	0.58	0.38	1.66 (0.53–5.10)
	Standard FST protocol	6	0.83 (0.68–0.92)	0.89 (0.85–0.93)	6.90 (4.72–10.08)	0.27 (0.17–0.42)	36.73 (17.65–76.44)				
Mixed outcome of RRT and AKI progression	Yes	2	0.92 (0.72–0.98)	0.94 (0.20–0.99)	3.08 (1.71–5.56)	0.15 (0.05–0.50)	25.98 (5.40–125.08)	0.15	0.86	0.86	1.16 (0.22–6.23)
	No	7	0.79 (0.71–0.85)	0.88 (0.83–0.91)	6.07 (4.45–8.29)	0.27 (0.20–0.38)	30.26 (16.67–54.94)				

*Abbreviation: AKI* acute kidney injury, *AKIN* Acute Kidney Injury Network, *KDIGO* Kidney Disease: Improving Global Outcomes, *LR* likelihood ratio, *NGAL* neutrophil gelatinase-associated lipocalin, *RDOR* relative diagnostic odd ratio, *SE* standard error, *UOP* urine output

Standard FST protocol (dose 1 mg/kg for furosemide naive or 1.5 mg/kg for furosemide non-naive patients and urine output cutoff value 200 ml within 2 h)

There were 2 studies that provided a composite outcome consisting of diagnostic performance of FST for AKI progression and RRT prediction (Elsaegh; Saber) [14, 20]. A sensitivity analysis was conducted after excluding these two trials. The pooled sensitivity and specificity values of the remaining 7 studies were 0.79 (95% CI 0.71–0.85) and 0.88 (95% CI 0.83–0.91), respectively. The pooled positive LR was 6.07 (95% CI 4.45–8.29), and the negative LR was 0.27 (95% CI 0.20–0.38) (Supplementary Figure 8). The pooled DOR was 30.26 (95% CI 16.67–54.94) (Supplementary Figure 9). The SROC with pooled diagnostic accuracy was 0.90 (Supplementary Figure 10).

We also performed Spearman rank correlations (*ρ* = 0.579; *P* = 0.23) and then subgroup analysis for FST as an RRT prediction tool. The RRT incidence is different in enrolled studies (from 15.6% in the study by Matsuura et al. to 66.6% in the study by Lumlertgul et al.). These six studies are also with variable follow-up period (from 1 day to 30 days) and enrolled patients of different AKI severity (stage 1–2 or stage 3). Subgroup analysis showed that the diagnostic performance was not affected by study population with different RRT incidences (RRT incidence < 20% vs. ≥ 20%; the relative diagnostic odds ratio 1.19 with 95% CI 0.37–3.78) or different follow-up durations (follow-up duration not reported or < 7 days vs. ≥ 7 days; the relative diagnostic odds ratio 3.71 with 95% CI 0.80–71.11). However, the diagnostic performance was better in early AKI stage population (stage 1–2) than in stage 3 (relative diagnostic odds ratio 5.75 with 95% CI 2.51–13.33) (Table 4).

**Table 4:** Heterogeneity analysis of meta-analyses (outcome included sensitivity analysis and meta-regression) for furosemide stress test as a renal replacement therapy prediction tool

Variables	Subgroups	Number of studies	Sensitivity (95% CI)	Specificity (95% CI)	Positive LR (95% CI)	Negative LR (95% CI)	Diagnostic odds ratio (95% CI)	Coeff.	SE	*P* value	RDOR (95% CI)
RRT rate	< 20%	2	0.80 (0.57–0.92)	0.82 (0.73–0.88)	4.35 (2.66–7.10)	0.25 (0.11–0.61)	17.52 (5.16–59.44)	0.17	0.59	0.77	1.19 (0.37–3.78)
	≥ 20%	4	0.85 (0.69–0.93)	0.76 (0.54–0.89)	2.64 (1.62–4.32)	0.25 (0.12–0.51)	12.81 (4.19–39.12)				
Follow-up duration	Not reported or < 7 days	3	0.80 (0.74–0.8 5)	0.74 (0.52–0.89)	2.89 (1.49–5.57)	0.37 (0.28–0.50)	4.97 (3.32–7.44)	1.31	0.78	0.09	3.71 (0.80–71.11)
	≥ 7 days	3	0.91 (0.66–0.98)	0.77 (0.65–0.86)	3.42 (2.41–4.86)	0.15 (0.07–0.34)	30.46 (13.13–70.66)				
AKI stage enrolled	Stage 3	1						1.75	0.43	< 0.01	5.75 (2.51–13.33)
	Stage 1 or 2	5	0.85 (0.68–0.94)	0.80 (0.73–0.86)	3.77 (2.80–5.09)	0.21 (0.11–0.41)	24.93 (12.45–49.91)				

*Abbreviation: AKI* acute kidney injury, *LR* likelihood ratio, *RDOR* relative diagnostic odds ratio, *RRT* renal replacement therapy, *SE* standard error, *UOP* urine output

## Discussion

Furosemide has been used for decades. Its pharmacodynamics, pharmacokinetics, and adverse effects are well described in patients with chronic kidney disease or nephrotic syndrome, but less data is available regarding its effects in AKI populations. Because of its low cost and availability, using diuretic response as a preserved renal functional marker has been proposed. In 1973, Baek et al. reported that the urinary free water excretion following intravenous furosemide administration could serve as a diagnostic tool for acute tubular necrosis (ATN) [38]. Pandit and colleagues found that, while on furosemide therapy, patients who had urine output less than 1200 ml 1 day after coronary artery bypass graft surgery were more likely to experience AKI, with a specificity of 97.93% [39]. It has been no one until 2013, Chawla et al. proposed a standard FST protocol, in which diuretic-naive patients receive 1 mg/kg of furosemide and patients who were exposed to furosemide within 7 days received 1.5 mg/kg of furosemide [8]. They use 200 ml urine output at 2 h after furosemide administration to serve as a cutoff value. In subjects with normal renal function or mild AKI, the infusion dose and creatinine clearance are major determinants of diuretic response [47, 48]. After AKI, several tubular function alterations could affect diuretic response, including a decrease of Na-K-Cl cotransporter 2 expression, Na-K-ATPase redistribution [49], and organic acid transporter mistargeting [50]. Therefore, the FST seems to provide a quick and easy method for the assessment of glomerular filtration and tubular damage. Despite this aforementioned role in diagnostics, furosemide is unlikely to reduce mortality or decrease the risk of RRT in AKI populations [51]. We thus performed this systematic review and meta-analysis to clarify the predictive value of the FST on AKI progression, the need for RRT, and in-hospital mortality. First, the analysis of the diagnostic accuracy of the FST for AKI progression yielded an AUROC of 0.88, with pooled sensitivity and specificity values of 0.81 and 0.88, respectively. Although there are no studies directly comparing the diagnostic accuracy of FST with other biomarkers, the AUROC of FST for AKI progression is not inferior to that of biomarkers, which ranged from 0.70 to 0.85 in previous reports [3, 52, 53]. The diagnostic performance of FST was not affected by whether the enrolled patients have high plasma NGAL or not. Koyner et al., by using the same cohort with Chawla et al., reported the AUROC of FST was higher than that of each biomarker alone. Compared to the overall cohort, the diagnostic accuracy of FST improved in patients with elevated biomarkers [32]. The aforementioned studies and our work imply that FST could serve as a simple risk triage tool combined with or without novel biomarker in early AKI patients.

Second, our work demonstrated that use of the FST as a tool for RRT prediction had an AUROC of 0.86, with high heterogeneity in regarding pooled diagnostic indices. The pooled specificity and positive LR values of the FST for RRT prediction were relatively low. The subgroup analysis showed that diagnostic performance is better in early AKI population. According to the study by Lumlertgul et al., 25% of the FST non-responder eventually did not undergo RRT because these patients did not meet the conventional criteria to start RRT. Lumlertgul et al. also demonstrated that in FST non-responders, whether early or late RRT initiation did not affect short-term mortality or renal recovery [15]. On the other hand, the FST responders are less likely to receive RRT. Matsuura et al. showed that only 5.6% (2/36) FST responders underwent RRT, whereas up to 40% (6/15) of FST non-responders requires RRT [17]. The major problems of RRT prediction lie in the optimal time for RRT initiation. Recently, several randomized controlled trials regarding the optimal timing of RRT initiation were published. The ELAIN trial enrolled KDIGO stage 2 AKI and demonstrated survival benefit from early initiation of RRT. This trial was criticized for its single center designs, the enrollment of post-surgery population, and some patients with significant fluid overload [54]. The AKIKI trial enrolled ICU patients with KDIGO stage 3 AKI and demonstrated no benefit with earlier RRT initiation in regard to 60-day mortality [55]. The IDEAL-ICU trial enrolled patients with septic shock who achieved a “failure” stage of AKI by RIFLE criteria but without life-threatening conditions, and found that there was no survival benefit with “early” RRT [56]. Despite these large trials, we still have no conclusive answers about the optimal timing to start RRT. A recent published meta-analysis demonstrated that early RRT may be beneficial for a shorter duration on mechanical ventilation. However, a watchful waiting strategy based on conventional indications for RRT initiation was generally safe in regard to all-cause mortality [57]. FST non-responsiveness alone might not be a good indicator for RRT initiation. We should also take clinical condition, patient’s demand, and residual renal capacity into consideration as suggested by Acute Disease Quality Initiative XVII conference (ADQI) [58]. Overall, because of the inconsistency of timing of RRT initiation, FST non-responsiveness is not a good predictor for RRT; nevertheless, FST responsiveness might serve as a negative predictor for RRT, especially in early AKI stage.

Our study had several limitations. First, the risks of bias in the investigated studies were not low because of the existence of non-prospective study design, inconsistent diagnostic cutoff values, and mixed patient populations. Second, the serum albumin level has been considered as a factor of diuretic resistance based on early experimental data [59], and recent studies have shown that the co-administration of albumin and loop diuretics might transiently increase urine water and sodium excretion [60, 61]. However, we did not have information about the serum albumin level in most studies and whether loop diuretics were co-administered with albumin in the enrolled studies. Third, the indications for RRT initiation were not precisely reported in most studies. Further prospective studies with standard RRT initiation protocol are needed for further evaluation the ability of FST for RRT prediction. Due to the lack of large prospective studies meeting our criteria for inclusion, the total number of enrolled patients was relatively small. Two completed but not published trial (NCT02730117, NCT04215419) and another ongoing trial (NCT 01275729) were identified in the process of systematic research. Further results from these larger clinical studies are required in the future for validation the diagnostic role of FST in AKI severity.

## Conclusion

In conclusion, FST non-responsiveness has a good predictive ability for AKI progression. The diagnostic performance of FST for RRT prediction is suboptimal and is better in early AKI population. Further trials with larger sample sizes with a high-quality study design are warranted to clarify the benefit of FST in the clinical setting.

## Supplementary information

**Additional file 1: Figure S1.** Diagnostic odd ratio of FST for prediction of AKI progression.

**Additional file 2: Figure S2.** SROC curves of FST for prediction of AKI progression, SROC (summary receiver operating characteristic).

**Additional file 3: Figure S3.** Diagnostic odd ratio of FST for prediction of RRT.

**Additional file 4: Figure S4.** SROC curves of FST for prediction of RRT.

**Additional file 5: Figure S5.** Forest plot of FST diagnostic accuracy for mortality prediction.

**Additional file 6: Figure S6.** Diagnostic odd ratio of FST for prediction of mortality.

**Additional file 7: Figure S7.** SROC curves of FST for prediction of mortality.

**Additional file 8: Figure S8.** Forest plot of FST diagnostic accuracy for AKI stage progression (exclusion of RRT).

**Additional file 9: Figure S9.** Diagnostic odd ratio of FST for prediction of AKI stage progression (exclusion of RRT).

**Additional file 10: Figure S10.** SROC curves of FST for prediction of AKI stage progression (exclusion of RRT).

**Additional file 11.** Supplemental Tables.

**Additional file 12.** Supplemental Document.

**Additional file 13.** Registered PROSPERO review protocol.

**Additional file 14.** GRADE Evidence and Summary of Findings Table.

## Data Availability

Not applicable.

## Abbreviations

AKI: Acute kidney injury
AKIN: Acute Kidney Injury Network
FST: Furosemide stress test
KDIGO: Kidney Disease: Improving Global Outcomes
NGAL: Neutrophil gelatinase-associated lipocalin
PC: Prospective cohort
RC: Retrospective cohort
