# Supplementary figures and images for: Furosemide stress test as a predictive marker of acute kidney injury progression or renal replacement therapy: a systemic review and meta-analysis
Source: Crit Care. 2020 May 7;24:202. doi: 10.1186/s13054-020-02912-8 (PMC7206785; doi:10.1186/s13054-020-02912-8)

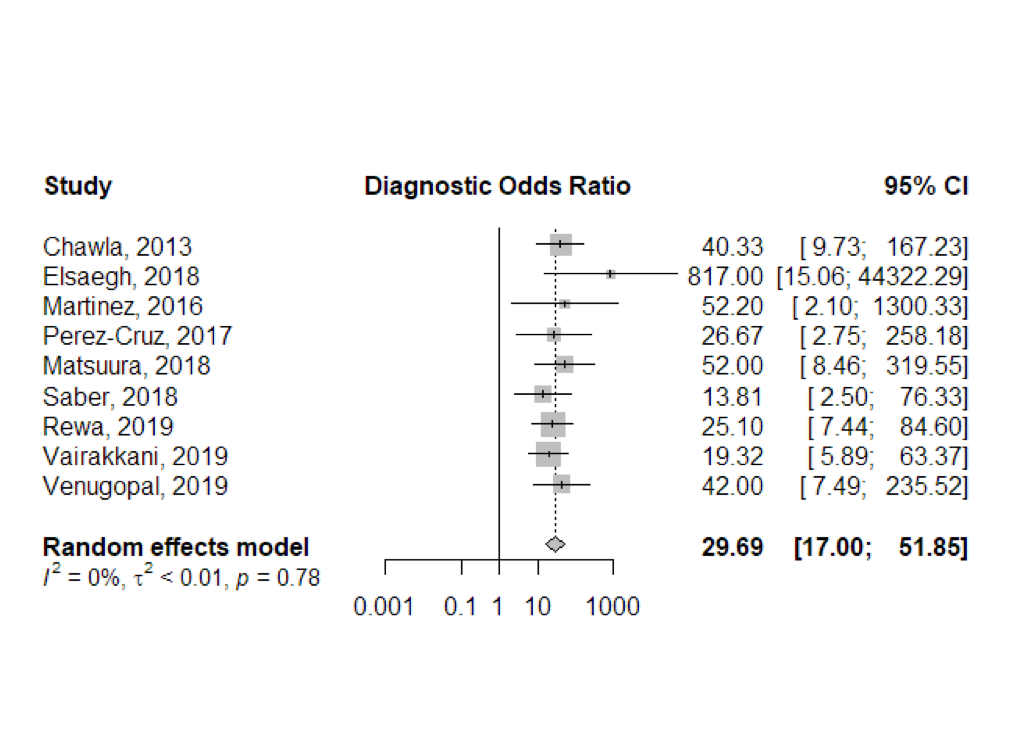

Supplement: Supplementary file 1 — Additional file 1: Figure S1. Diagnostic odd ratio of FST for prediction of AKI progression. [file 13054_2020_2912_MOESM1_ESM.tif]

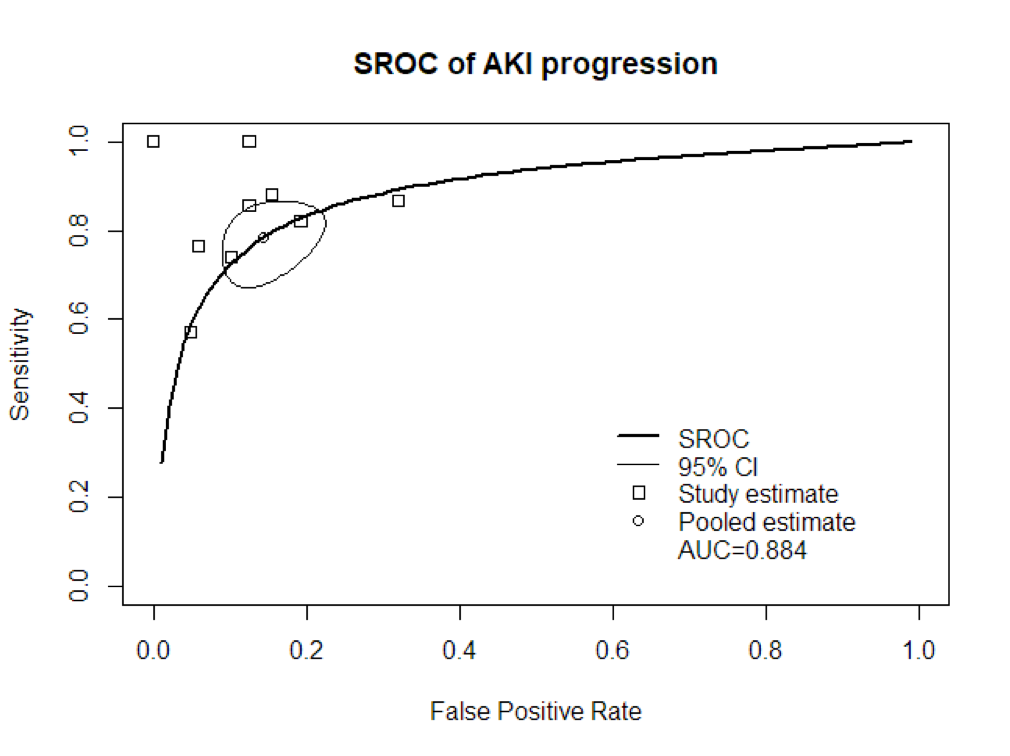

Supplement: Supplementary file 2 — Additional file 2: Figure S2. SROC curves of FST for prediction of AKI progression, SROC (summary receiver operating characteristic). [file 13054_2020_2912_MOESM2_ESM.tif]

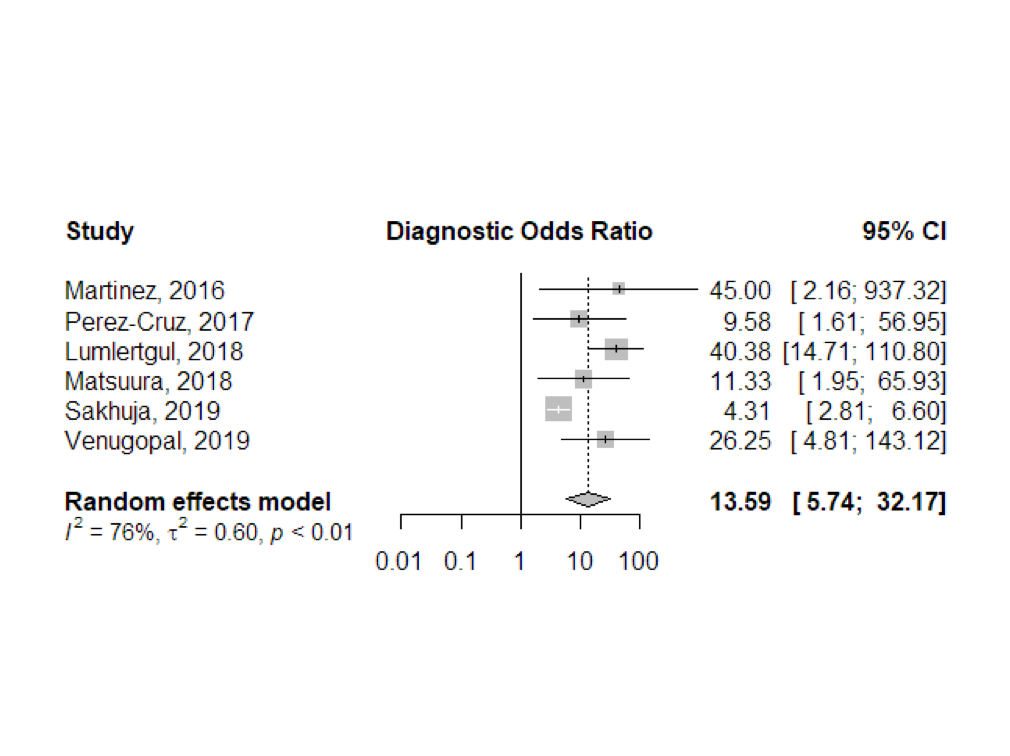

Supplement: Supplementary file 3 — Additional file 3: Figure S3. Diagnostic odd ratio of FST for prediction of RRT. [file 13054_2020_2912_MOESM3_ESM.tif]

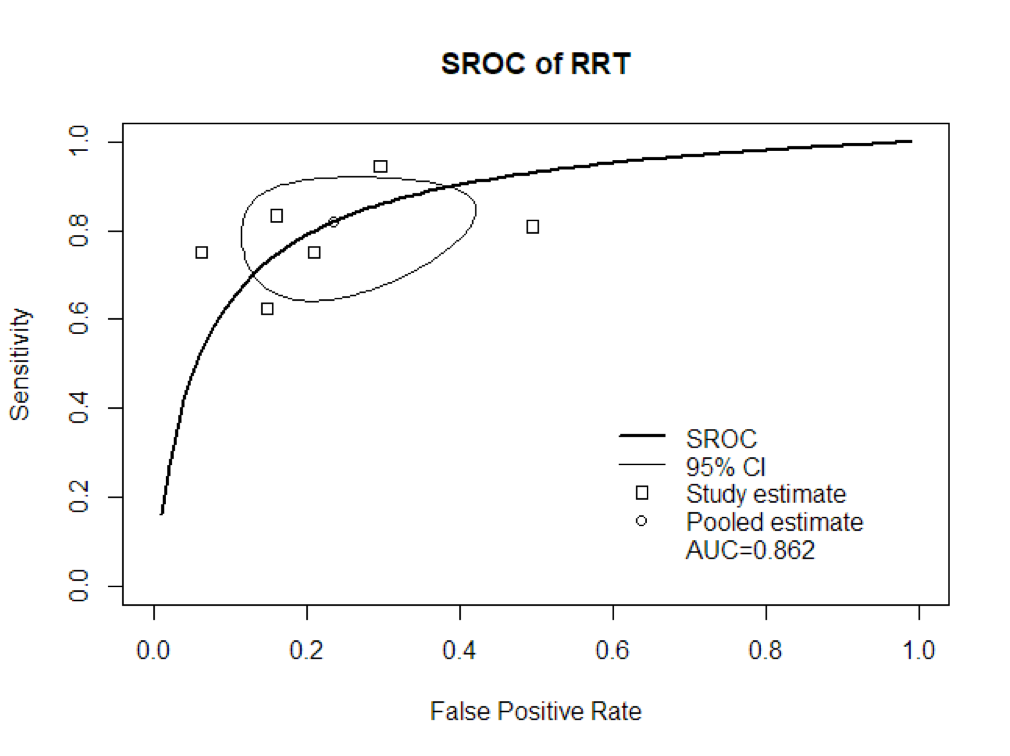

Supplement: Supplementary file 4 — Additional file 4: Figure S4. SROC curves of FST for prediction of RRT. [file 13054_2020_2912_MOESM4_ESM.tif]

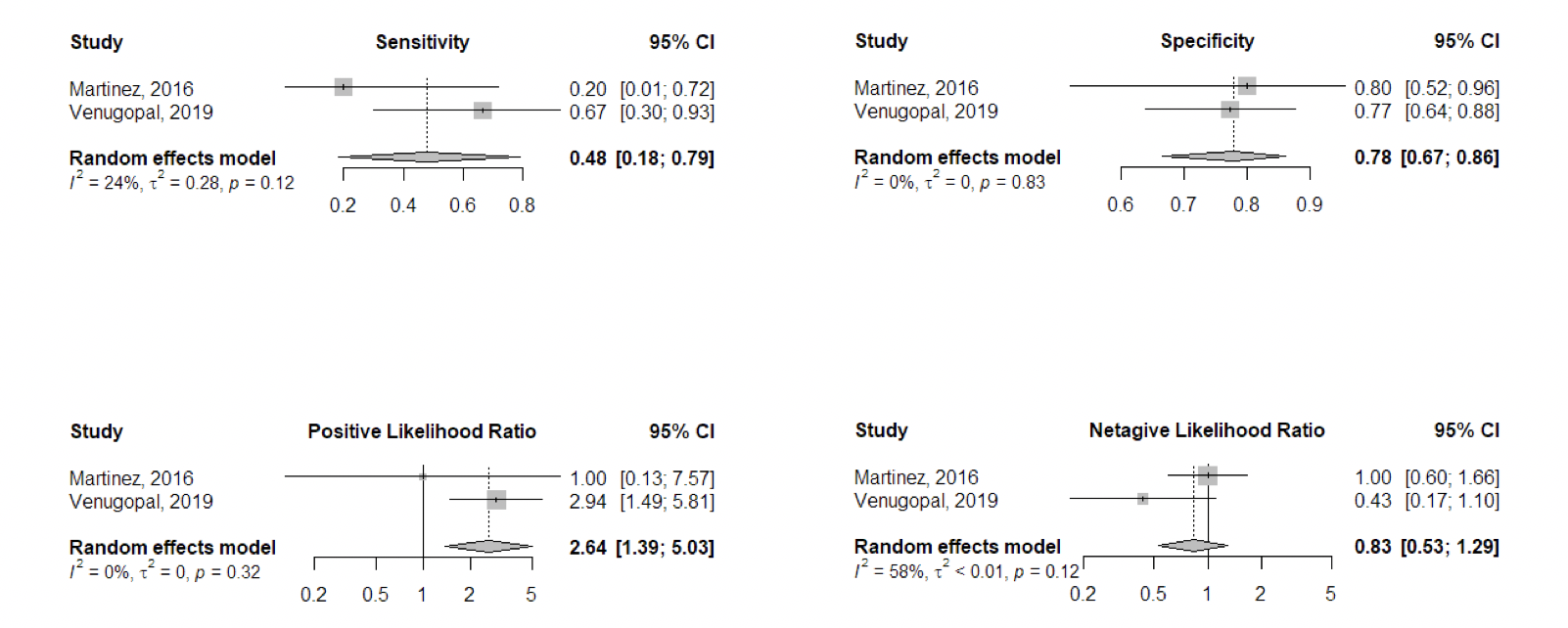

Supplement: Supplementary file 5 — Additional file 5: Figure S5. Forest plot of FST diagnostic accuracy for mortality prediction. [file 13054_2020_2912_MOESM5_ESM.tif]

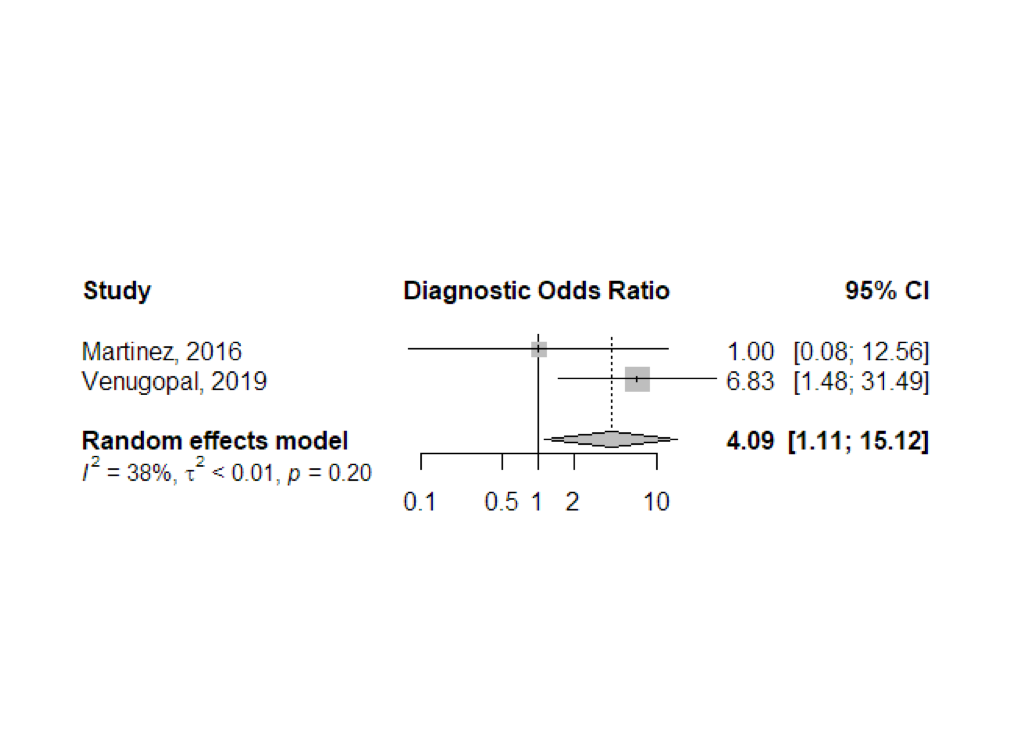

Supplement: Supplementary file 6 — Additional file 6: Figure S6. Diagnostic odd ratio of FST for prediction of mortality. [file 13054_2020_2912_MOESM6_ESM.tif]

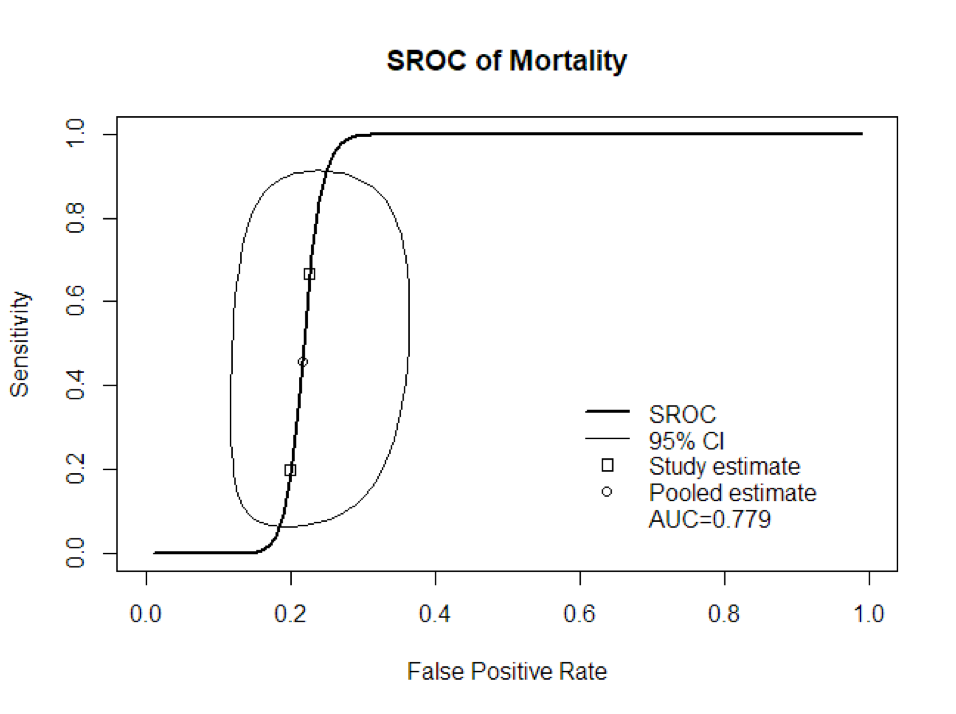

Supplement: Supplementary file 7 — Additional file 7: Figure S7. SROC curves of FST for prediction of mortality. [file 13054_2020_2912_MOESM7_ESM.tif]

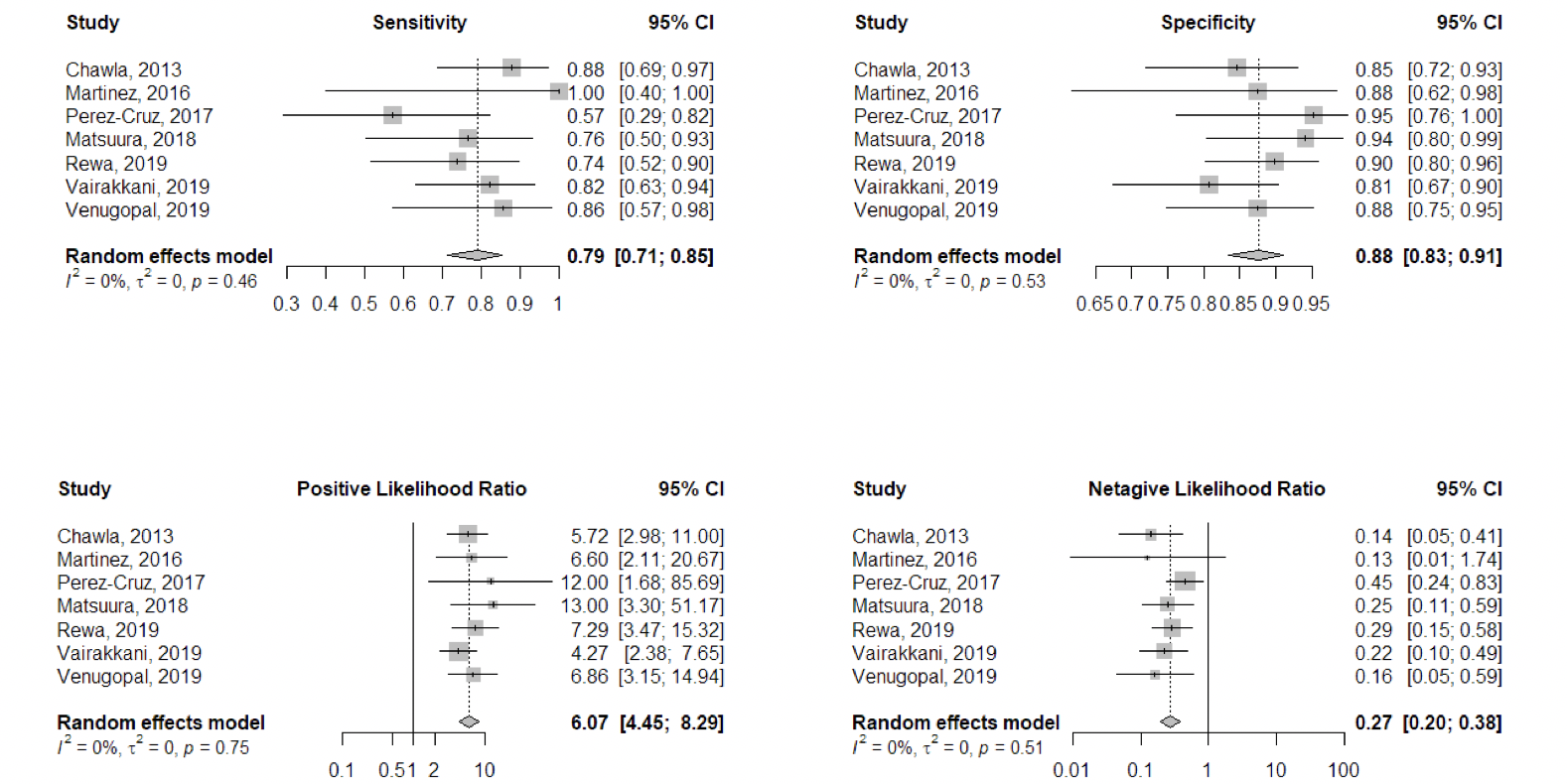

Supplement: Supplementary file 8 — Additional file 8: Figure S8. Forest plot of FST diagnostic accuracy for AKI stage progression (exclusion of RRT). [file 13054_2020_2912_MOESM8_ESM.tif]

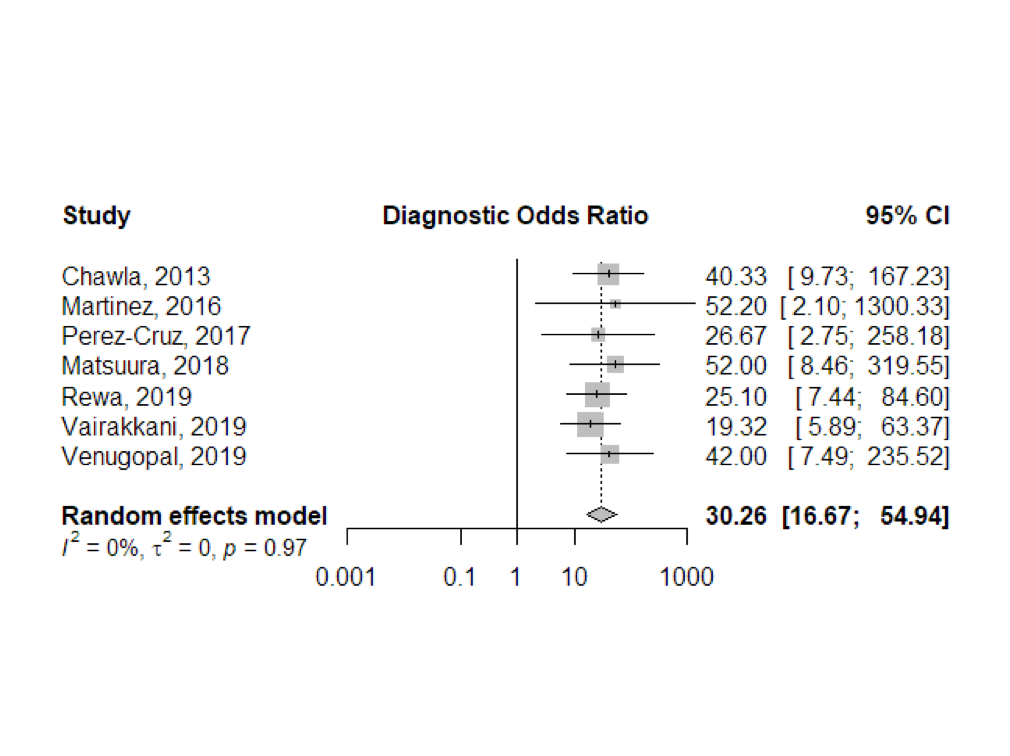

Supplement: Supplementary file 9 — Additional file 9: Figure S9. Diagnostic odd ratio of FST for prediction of AKI stage progression (exclusion of RRT). [file 13054_2020_2912_MOESM9_ESM.tif]

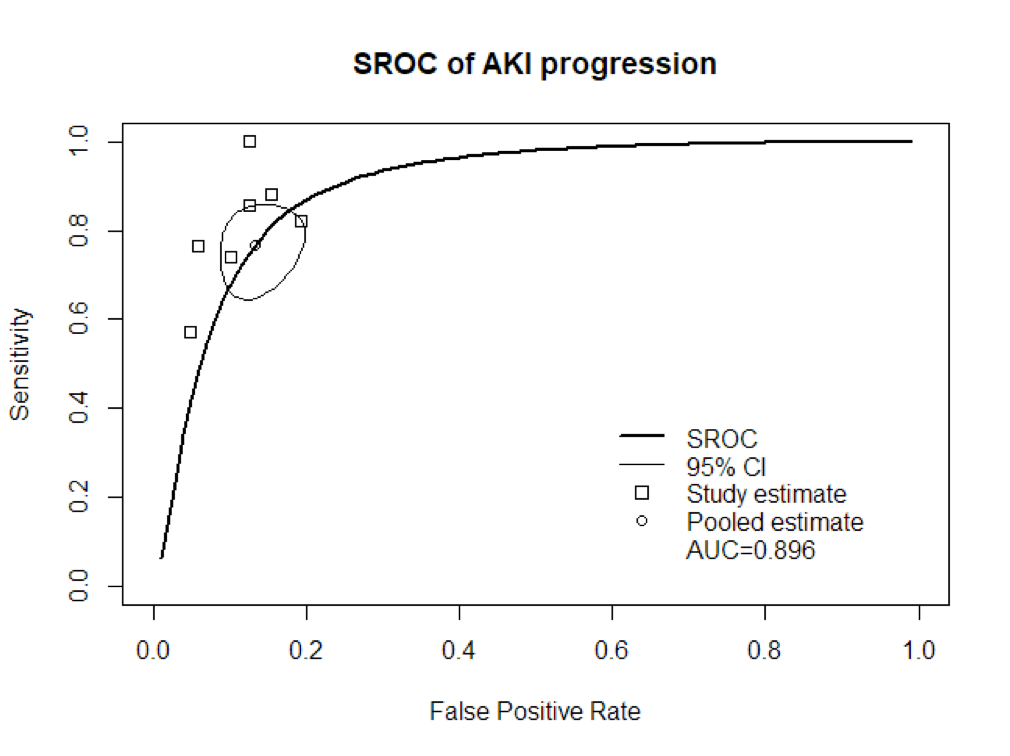

Supplement: Supplementary file 10 — Additional file 10: Figure S10. SROC curves of FST for prediction of AKI stage progression (exclusion of RRT). [file 13054_2020_2912_MOESM10_ESM.tif]
