# Supplementary material for: Furosemide stress test as a predictive marker of acute kidney injury progression or renal replacement therapy: a systemic review and meta-analysis
Source: Crit Care. 2020 May 7;24:202. doi: 10.1186/s13054-020-02912-8 (PMC7206785; doi:10.1186/s13054-020-02912-8)
Supplement: Supplementary file 11 — Additional file 11. Supplemental Tables. [file 13054_2020_2912_MOESM11_ESM.docx]

**Supplementary Table 1A**

**Details of Search Strategy Source: PubMed; Searched on: 13, Mar, 2020; Results: 1338**

| **Search** | **Query** | **Items** |
| --- | --- | --- |
| [#1](https://www.ncbi.nlm.nih.gov/pubmed/advanced) | "furosemide"[Mesh] | [11786](https://www.ncbi.nlm.nih.gov/pubmed/?cmd=HistorySearch&querykey=10) |
| #2 | "lasix"[Title/Abstract] | 330 |
| #3 | "furosemide"[Title/Abstract] | 11752 |
| **#4** | [**#1**](https://www.ncbi.nlm.nih.gov/pubmed/advanced) **OR** [**#2**](https://www.ncbi.nlm.nih.gov/pubmed/advanced) **OR #3** | **16667** |
| #5 | "Renal Insufficiency"[Mesh] | 169181 |
| #6 | "Renal insufficiency"[Title/Abstract] | 22806 |
| #7 | "Acute Kidney Injury"[Mesh] | 45260 |
| #8 | "Acute kidney injury"[Title/Abstract] | 21597 |
| #9 | AKI[Title/Abstract] | 11858 |
| **#10** | **#5 OR #6 OR #7 OR #8 OR #9** | **189668** |
| **#11** | **#4 AND #10** | **1338** |

**Supplementary Table 1B**

**Details of Search Strategy Source: Embase; Searched on: 13, Mar, 2020; Results: 556**

| **Search** | **Query** | **Items** |
| --- | --- | --- |
| [#1](https://www.ncbi.nlm.nih.gov/pubmed/advanced) | furosemide:ab,ti OR lasix:ab,ti | [17609](https://www.ncbi.nlm.nih.gov/pubmed/?cmd=HistorySearch&querykey=10) |
| #2 | 'acute kidney injury':ab,ti OR 'acute renal failure':ab,ti | 64159 |
| #3 | [**#1**](https://www.ncbi.nlm.nih.gov/pubmed/advanced) **OR** [**#2**](https://www.ncbi.nlm.nih.gov/pubmed/advanced) | **711** |
| #4 | **#1 AND #2 AND [humans]/lim** | **556** |

**Supplementary Table 2 Primary reasons for exclusion of excluded studies**

| **First Author** | **Last Author** | **Journal** | **Year** | **Title** | **Primary Reason for Exclusion** | **Include** |
| --- | --- | --- | --- | --- | --- | --- |
| Aadil Kakajiwala | Joshua J. Blinder | Ann Thorac Surg | 2017 | Lack of Furosemide Responsiveness Predicts Acute Kidney Injury in Infants After Cardiac Surgery | Not adult population | N |
| Amrita S Pandit | Eugene Fernandes | Journal of the American College of Surgeons | 2011 | Response to furosemide as marker of acute kidney injury in post-operative CABG patients | Unclear AKI definition, no furosemide dose | N |
| Blaithin A. McMahon | Edward Kraus | Biomarker | 2017 | The prognostic value of the furosemide stress test in predicting delayed graft function following deceased donor kidney transplantation | Kidney transplant population | N |
| Davison D.L | Chawla L.S. | American Journal of Kidney Diseases | 2014 | The furosemide stress test in combination with urinary biomarkers to predict the progression and severity of acute kidney injury | Duplication cohort | N |
| H. Arifianto | B. Purwanto | European Heart Journal Supplements | 2017 | Acute kidney injury diagnosis in acute heart failure, does furosemide stress test make sense? | Insufficiency information | N |
| Jay L. Koyner | Lakhmir S. Chawla | J Am Soc Nephrol | 2015 | Furosemide Stress Test and Biomarkers for the Prediction of AKI Severity | Duplication cohort | N |
| Jesús Rivero | Magdalena Madero | BMC Nephrol | 2020 | Furosemide Stress Test and Interstitial Fibrosis in Kidney Biopsies in Chronic Kidney Disease | Different outcome of interest | N |
| J. Kataoka | S. Fujitani | Intensive Care Medicine Experimental | 2017 | Does the response in urine output to a small dose of furosemide predict organ failure after achievement of negative fluid balance in acute respiratory failure? The interim analysis | Different outcome of interest | N |
| Palma l | Perez R | American Journal of Transplantation | 2017 | The use of a furosemide stress test (fst) for assessment of discarded deceased donor kidneys in an ex-vivo normothermic perfusion model | Kidney transplant population | N |
| Penk Jamie | Rajit K. Basu | Journal of Thoracic and Cardiovascular Surgery | 2019 | Furosemide response predicts acute kidney injury in children after cardiac surgery | Not adult population | N |
| Peter HJ van der Voort. | Peter Pickkers | Critical Care | 2014 | The furosemide stress test to predict renal function after continuous renal replacement therapy | Different outcome of interest | N |
| R Vargas | E López | Bol Med Hosp Infant Mex | 1977 | Furosemide in the Early Diagnosis of Acute Renal Insufficiency in the Newborn Infant | Not adult population | N |
| Rivera SG | Calyeca SMV | Med Crit | 2018 | Furosemide stress test to predict success or failure to remove continuos slow renal replacement therapy in acute renal injury | Different outcome of interest | N |
| Santiago Borasino | Jeffrey A. Alten | Pediatr Crit Care Med | 2018 | Furosemide Response Predicts Acute Kidney Injury After Cardiac Surgery in Infants and Neonates | Not adult population | N |
| S. M. Baek | W. C. Shoemaker | Ann Surg | 1973 | Early prediction of acute renal failure and recovery. II. Renal function response to furosemide | No urine output cutoff point, unclear AKI definition | N |
| Suprita Kalra | R K Gupta | Indian J Child Health | 2017 | Use of  furosemide stress test for edema control and predicting acute kidney injury in children with nephrotic syndrome | Not adult population | N |
| Suwasin Udomkarnjananun | Yingyos Avihingsanon | Nephron | 2019 | Furosemide Stress Test as a Predicting Biomarker for Delayed Graft Function in Kidney Transplantation | Kidney transplant population | N |
| V V Arkhipov | O V Levicheva | - Klin Lab Diagn | 2011 | Functional Furosemide Loading Test. Practical Use in Children With Kidney Diseases | Different outcome of interest | N |

**Supplementary Table 3 The other characteristics of the included studies**

| First author/ year | Pre-specific cot-off value | Report of serum albumin level | Report of renal replacement therapy initiation indication |
| --- | --- | --- | --- |
| Chawla, 2013 | Yes | No | NA |
| Elsaegh, 2018 | Yes | No | No |
| Lumlertgul, 2018 | Yes | Exclusion of patients with serum albumin level < 2 g/dL | Yes # |
| Martínez, 2016 | Yes | No | No |
| Matsuura, 2018 | No | 2.8 g/dL | No |
| Pérez-Cruz, 2017 | Yes | No | No |
| Rewa, 2019 | No | No | NA |
| Saber, 2018 | No | No | No |
| Sakhuja, 2019 | No | 2.9 g/dL | No |
| Vairakkani, 2019 | No | No | NA |
| Venugopal, 2019 | Yes | No | No |

**Abbreviation:** NA (not applicable)

# Patient received RRT within 6 hours after randomization in early group or received RRT based on conventional indications in standard group
