# Supplementary material for: Furosemide stress test as a predictive marker of acute kidney injury progression or renal replacement therapy: a systemic review and meta-analysis
Source: Crit Care. 2020 May 7;24:202. doi: 10.1186/s13054-020-02912-8 (PMC7206785; doi:10.1186/s13054-020-02912-8)
Supplement: Supplementary file 12 — Additional file 12. Supplemental Document. [file 13054_2020_2912_MOESM12_ESM.docx]

**Supplemental Document**

***Grey Literature Search***

For searching grey literature, we did not set article type or language filter in Embase. We also used keyword "Furosemide" combined with "Acute Kidney Injury" or "furosemide stress test" in google scholar, ResearchGate and other relative online resources mentioned in Cochrane Handbook and in “Grey Matters: a practical tool for searching health-related grey literature”. In accordance with Cochrane handbook’s suggestion, we checked grey literature from the OpenGrey and the National Technical Information Service (NTIS) with keyword “Acute kidney injury” and “Furosemide”. Some newly found relative articles from above mentioned resources but not fulfilled enrolled criteria are summarized into **Additional profile 2: Supplemental Table 2**. We also checked unpublished or ongoing clinical trial and three related studies were identified (NCT02730117, NCT04215419, NCT 01275729). After our search of grey literatures, further two studies were included in our final analysis (Martínez, 2016; Pérez-Cruz, 2017), whereas others were not eligible because the information was insufficient for analysis or different outcome of interest.
