# Supplementary material for: Furosemide stress test as a predictive marker of acute kidney injury progression or renal replacement therapy: a systemic review and meta-analysis
Source: Crit Care. 2020 May 7;24:202. doi: 10.1186/s13054-020-02912-8 (PMC7206785; doi:10.1186/s13054-020-02912-8)
Supplement: Supplementary file 13 — Additional file 13. Registered PROSPERO review protocol. [file 13054_2020_2912_MOESM13_ESM.pdf]

Systematic review

This record cannot be edited because it is being assessed by the editorial team

1. * Review title.

Give the working title of the review, for example the one used for obtaining funding. Ideally the title should state succinctly the interventions or exposures being reviewed and the associated health or social problems. Where appropriate, the title should use the PI(E)COS structure to contain information on the Participants, Intervention (or Exposure) and Comparison groups, the Outcomes to be measured and Study designs to be included.

Role of Furosemide Stress test in acute kidney injury severity and need for renal replacement therapy prediction : systemic review and meta-analysis

2. Original language title.

For reviews in languages other than English, this field should be used to enter the title in the language of the review. This will be displayed together with the English language title.

3. * Anticipated or actual start date.

Give the date when the systematic review commenced, or is expected to commence.

11/12/2019

4. * Anticipated completion date.

Give the date by which the review is expected to be completed.

29/02/2020

5. * Stage of review at time of this submission.

Indicate the stage of progress of the review by ticking the relevant Started and Completed boxes. Additional information may be added in the free text box provided.

Please note: Reviews that have progressed beyond the point of completing data extraction at the time of initial registration are not eligible for inclusion in PROSPERO. Should evidence of incorrect status and/or completion date being supplied at the time of submission come to light, the content of the PROSPERO record will be removed leaving only the title and named contact details and a statement that inaccuracies in the stage of the review date had been identified.

This field should be updated when any amendments are made to a published record and on completion and publication of the review. If this field was pre-populated from the initial screening questions then you are not able to edit it until the record is published.

The review has not yet started: No

Review stage	Started	Completed
Preliminary searches	Yes	No
Piloting of the study selection process	Yes	No
Formal screening of search results against eligibility criteria	Yes	No
Data extraction	No	No
Risk of bias (quality) assessment	No	No

Review stage	Started	Completed
Data analysis	No	No

Provide any other relevant information about the stage of the review here (e.g. Funded proposal, protocol not yet finalised).

6. * Named contact.

The named contact acts as the guarantor for the accuracy of the information presented in the register record.

Jia-Jin Chen,

Email salutation (e.g. "Dr Smith" or "Joanne") for correspondence:
Dr Chen

7. * Named contact email.

Give the electronic mail address of the named contact.

raymond110234@hotmail.com

8. Named contact address

PLEASE NOTE this information will be published in the PROSPERO record so please do not enter private information

Give the full postal address for the named contact.

No 5 Fu-shin street, Taoyuan 333, Taiwan

9. Named contact phone number.

Give the telephone number for the named contact, including international dialling code.

886-3-3281200 ext 8181

10. * Organisational affiliation of the review.

Full title of the organisational affiliations for this review and website address if available. This field may be completed as 'None' if the review is not affiliated to any organisation.

Division of nephrology, Linkou Chang Gung Memorial hospital, Taiwan

Organisation web address:

11. * Review team members and their organisational affiliations.

Give the personal details and the organisational affiliations of each member of the review team. Affiliation refers to groups or organisations to which review team members belong. **NOTE: email and country are now mandatory fields for each person.**

Dr Jia-Jin Chen. Division of nephrology, Linkou Chang Gung Memorial hospital, Taiwan
George Kuo. Division of nephrology, Linkou Chang Gung Memorial hospital, Taiwan

12. * Funding sources/sponsors.

Give details of the individuals, organizations, groups or other legal entities who take responsibility for initiating, managing, sponsoring and/or financing the review. Include any unique identification numbers assigned to the review by the individuals or bodies listed.

no

Grant number(s)

13. * Conflicts of interest.

List any conditions that could lead to actual or perceived undue influence on judgements concerning the main topic investigated in the review.

None

14. Collaborators.

Give the name and affiliation of any individuals or organisations who are working on the review but who are not listed as review team members. **NOTE: email and country are now mandatory fields for each person.**

15. * Review question.

State the question(s) to be addressed by the review, clearly and precisely. Review questions may be specific or broad. It may be appropriate to break very broad questions down into a series of related more specific questions. Questions may be framed or refined using PI(E)COS where relevant.

What is the prognosis triage role of furosemide stress test in acute kidney injury ?

16. * Searches.

State the sources that will be searched. Give the search dates, and any restrictions (e.g. language or publication period). Do NOT enter the full search strategy (it may be provided as a link or attachment.)

PubMed , Embase, MEDLINE–ovid, Cochrane Library , search date: 2019/12/05 , language: English, publication peroid: no limitation

17. URL to search strategy.

Give a link to a published pdf/word document detailing either the search strategy or an example of a search strategy for a specific database if available (including the keywords that will be used in the search strategies), or upload your search strategy.

Do NOT provide links to your search results.

Do not make this file publicly available until the review is complete

18. * Condition or domain being studied.

Give a short description of the disease, condition or healthcare domain being studied. This could include health and wellbeing outcomes.

acute kidney injury

19. * Participants/population.

Give summary criteria for the participants or populations being studied by the review. The preferred format includes details of both inclusion and exclusion criteria.

Inclusion: Adult kidney injury in adult (diagnosis by either AKIN, RIFLE or KDIGO criteria)
Exclusion: transplant kidney, child (younger than 18 years old)

20. * Intervention(s), exposure(s).

Give full and clear descriptions or definitions of the nature of the interventions or the exposures to be reviewed.

Furosemide stress test

21. * Comparator(s)/control.

Where relevant, give details of the alternatives against which the main subject/topic of the review will be compared (e.g. another intervention or a non-exposed control group). The preferred format includes details of both inclusion and exclusion criteria.

nil

22. * Types of study to be included.

Give details of the types of study (study designs) eligible for inclusion in the review. If there are no restrictions on the types of study design eligible for inclusion, or certain study types are excluded, this should be stated. The preferred format includes details of both inclusion and exclusion criteria.

prospective and retrospective study

23. Context.

Give summary details of the setting and other relevant characteristics which help define the inclusion or exclusion criteria.

24. * Main outcome(s).

Give the pre-specified main (most important) outcomes of the review, including details of how the outcome is defined and measured and when these measurement are made, if these are part of the review inclusion criteria.

acute kidney injury stage progression or need for renal replacement therapy

* Measures of effect

1. AKI stage progression (from stage 2 to stage 3 by KDIGO or AKIN or RIFLE criteria)
2. Need for renal replacement therapy (dependent on physician or pre-defined RRT initiation criteria)

25. * Additional outcome(s).

List the pre-specified additional outcomes of the review, with a similar level of detail to that required for main outcomes. Where there are no additional outcomes please state ‘None’ or ‘Not applicable’ as appropriate to the review

mortality

* Measures of effect

all cause mortality in hospital

26. * Data extraction (selection and coding).

Describe how studies will be selected for inclusion. State what data will be extracted or obtained. State how this will be done and recorded.

Data of study characteristics that were extracted included first author, year of publication, study location, study design, acute kidney injury criteria, inclusion and exclusion criteria, patient number (number of total population), dose of furosemide, definition of furosemide stress test response. Data of patient characteristics included male, age, diabetes, hypertension, patient population (surgical, medical, ICU), creatinine at AKI diagnosis , AKI stage when enrolled, urine output after furosemide in both responder and non-responder group, peak AKI stage. Items related to diagnostic test performance were also extracted, including sensitivity, specificity, and the number of AKI stage progression and/or number of need for RRT after AKI

27. * Risk of bias (quality) assessment.

Describe the method of assessing risk of bias or quality assessment. State which characteristics of the studies will be assessed and any formal risk of bias tools that will be used.

We used the Quality Assessment of Diagnostic Accuracy Studies 2 (QUADAS-2) tool to assess the quality of the included studies. Disagreements between the two reviewers were solved by consensus through discussion. If the

answer to all signaling questions in each domain is “yes”, the domain is considered as low risk. If any signaling question is answered “no”, the domain is considered as a high risk of bias.

28. * Strategy for data synthesis.

Provide details of the planned synthesis including a rationale for the methods selected. This **must not be generic text** but should be **specific to your review** and describe how the proposed analysis will be applied to your data.

True positive (TP), true negative (TN), false positive (FP), and false negative (FN) rates for each study were calculated according to the reported sensitivity, specificity and patient number of AKI stage progression or need for RRT. Based on these data, positive likelihood ratio (+LR), negative likelihood ratio (–LR), and diagnostic odds ratio (DOR) could be obtained for each study. The summary measures were calculated using a random effects model (DerSimonian and Laird method). To assess the diagnostic performance of furosemide stress test in predicting AKI progression or need for RRT, a summary receiver operating characteristics (SROC) curve was constructed based on TP and FP rates. The threshold effect was detected using the Spearman correlation coefficient between the logit of sensitivity and logit of ‘1 — specificity’, where a non-significant threshold effect was warranted before performing further subgroup analysis or meta-regression. The degree of heterogeneity among studies was evaluated using the I^2 index, with < 25%, 25% – 50%, and > 50% indicating mild, moderate, and high heterogeneity, respectively.

29. * Analysis of subgroups or subsets.

State any planned investigation of ‘subgroups’. Be clear and specific about which type of study or participant will be included in each group or covariate investigated. State the planned analytic approach.

Possible sources of heterogeneity was examining by sensitivity analyses according to: population characteristics (severity of AKI when enrolled, patient criteria), study design (prospective or retrospective), definition of furosemide stress test non-responder, dose of prescribed furosemide, acute kidney injury criteria

30. * Type and method of review.

Select the type of review and the review method from the lists below. Select the health area(s) of interest for your review.

Type of review

Cost effectiveness	No
Diagnostic	Yes
Epidemiologic	No
Individual patient data (IPD) meta-analysis	No
Intervention	No
Meta-analysis	No
Methodology	No
Narrative synthesis	No
Network meta-analysis	No
Pre-clinical	No
Prevention	No
Prognostic	No
Prospective meta-analysis (PMA)	No
Review of reviews	No
Service delivery	No

Synthesis of qualitative studies	No
Systematic review	Yes
Other	No

Health area of the review

Alcohol/substance misuse/abuse	No
Blood and immune system	No
Cancer	No
Cardiovascular	No
Care of the elderly	No
Child health	No
Complementary therapies	No
Crime and justice	No
Dental	No
Digestive system	No
Ear, nose and throat	No
Education	No
Endocrine and metabolic disorders	No
Eye disorders	No
General interest	No
Genetics	No
Health inequalities/health equity	No
Infections and infestations	No
International development	No
Mental health and behavioural conditions	No
Musculoskeletal	No
Neurological	No
Nursing	No
Obstetrics and gynaecology	No

Oral health	No
Palliative care	No
Perioperative care	No
Physiotherapy	No
Pregnancy and childbirth	No
Public health (including social determinants of health)	No
Rehabilitation	No
Respiratory disorders	No
Service delivery	No
Skin disorders	No
Social care	No
Surgery	No
Tropical Medicine	No
Urological	No
Wounds, injuries and accidents	No
Violence and abuse	No

31. Language.

Select each language individually to add it to the list below, use the bin icon to remove any added in error.

English

There is not an English language summary

32. * Country.

Select the country in which the review is being carried out from the drop down list. For multi-national collaborations select all the countries involved.

Taiwan

33. Other registration details.

Give the name of any organisation where the systematic review title or protocol is registered (such as with The Campbell Collaboration, or The Joanna Briggs Institute) together with any unique identification number assigned. (N.B. Registration details for Cochrane protocols will be automatically entered). If extracted data will be stored and made available through a repository such as the Systematic Review Data Repository (SRDR), details and a link should be included here. If none, leave blank.

34. Reference and/or URL for published protocol.

Give the citation and link for the published protocol, if there is one

No I do not make this file publicly available until the review is complete

35. Dissemination plans.

Give brief details of plans for communicating essential messages from the review to the appropriate audiences.

Do you intend to publish the review on completion?

Yes

36. Keywords.

Give words or phrases that best describe the review. Separate keywords with a semicolon or new line. Keywords will help users find the review in the Register (the words do not appear in the public record but are included in searches). Be as specific and precise as possible. Avoid acronyms and abbreviations unless these are in wide use.

37. Details of any existing review of the same topic by the same authors.

Give details of earlier versions of the systematic review if an update of an existing review is being registered, including full bibliographic reference if possible.

38. * Current review status.

Review status should be updated when the review is completed and when it is published. For newregistrations the review must be Ongoing.

Review_Ongoing

39. Any additional information.

Provide any other information the review team feel is relevant to the registration of the review.

40. Details of final report/publication(s).

This field should be left empty until details of the completed review are available.
