# Supplementary material for: Furosemide stress test as a predictive marker of acute kidney injury progression or renal replacement therapy: a systemic review and meta-analysis
Source: Crit Care. 2020 May 7;24:202. doi: 10.1186/s13054-020-02912-8 (PMC7206785; doi:10.1186/s13054-020-02912-8)
Supplement: Supplementary file 14 — Additional file 14. GRADE Evidence and Summary of Findings Table. [file 13054_2020_2912_MOESM14_ESM.pdf]

Question: Should Furosemide stress test be used to diagnose acute kidney injury progression in cases of suspected or clinical diagnosis acute kidney injury population?

Sensitivity	0.81 (95% CI: 0.74 to 0.87)
Specificity	0.88 (95% CI: 0.82 to 0.92)

Prevalences	10%	25%	50%

Outcome	№ of studies (№ of patients)	Study design	Factors that may decrease certainty of evidence					Effect per 1,000 patients tested			Test accuracy CoE
			Risk of bias	Indirectness	Inconsistency	Imprecision	Publication bias	pre-test probability of 10%	pre-test probability of 25%	pre-test probability of 50%	
**True positives** (patients with acute kidney injury progression)	9 studies 181 patients	cohort & case-control type studies	serious [a]	not serious	not serious	not serious	none	81 (74 to 87)	203 (185 to 218)	405 (370 to 435)	⨁⨁⨁◯ MODERATE
**False negatives** (patients incorrectly classified as not having acute kidney injury progression)								19 (13 to 26)	47 (32 to 65)	95 (65 to 130)	
**True negatives** (patients without acute kidney injury progression)	9 studies 336 patients	cohort & case-control type studies	serious [a]	not serious	not serious [b]	not serious	none	792 (738 to 828)	660 (615 to 690)	440 (410 to 460)	⨁⨁⨁◯ MODERATE
**False positives** (patients incorrectly classified as having acute kidney injury progression)								108 (72 to 162)	90 (60 to 135)	60 (40 to 90)	

Explanations

a. Most study did not used pre-specified threshold which might result in overestimating the diagnosis performance
b. The heterogeneity of pooled specificity: 42
